# Cardiac alterations induced by *Trypanosoma cruzi* extracellular vesicles and immune complexes

**DOI:** 10.1371/journal.pntd.0013273

**Published:** 2025-07-07

**Authors:** Alberto Cornet-Gomez, Francisco O’Valle, José M. Garrido, Fernando Rodríguez Serrano, Ana I. Nieto, Antonio Osuna

**Affiliations:** 1 Department of Parasitology, Biochemical and Molecular Parasitology Group CTS-183, and Institute of Biotechnology, University of Granada, Granada, Spain; 2 Instituto de Investigación Biosanitaria de Granada (ibs.GRANADA), Granada, Spain; 3 Department of Pathology, School of Medicine, University of Granada, Granada, Spain; 4 Biopathology and Regenerative Medicine Institute (IBIMER), University of Granada, Granada, Spain; 5 Department of Surgery and Surgical Specialties, School of Medicine, University of Granada, Granada, Spain; 6 Department of Human Anatomy and Embryology, University of Granada, Granada, Spain; 7 Centro de Investigación Biomédica, Universidad de Granada, Granada, Spain; Centro de Pesquisa Gonçalo Moniz-FIOCRUZ/BA, BRAZIL

## Abstract

**Background:**

*Trypanosoma cruzi* is a protozoan parasite responsible for American trypanosomiasis or Chagas disease (CD). This disease is characterized by the presence of cardiac or gastrointestinal symptoms in many patients during the chronic phase, with cardiac symptoms being the most common and severe, affecting approximately 30% of all patients. Although the origin of these pathologies remains unclear, several mechanisms have been proposed, involving factors related to *T. cruzi* and the host immune response. Extracellular vesicles (EVs) have been studied for their role in parasite-host cell communication, in modulating the host’s immune response and more recently as diagnostic tools.

**Methodology and main findings:**

In this study, we describe the role of EVs released by trypomastigotes and the immune complexes (ICs) they form with anti-*T. cruzi* IgGs (EVs-IgG) in the development of cardiac symptoms compatible with Chagas cardiomyopathy in mice. Autoantibodies detection, electrocardiographic, histopathological, and immunological analyses in mice’s hearts were performed.

The studies carried out revealed that, while the inoculation of EVs and ICs (seven intravenous injections of 2 µg of EVs and ICs over 21 days) did not elicit the appearance of autoantibodies, it led to ECG alterations (heart rate and PR interval), changes in heart cavity areas and wall thickness, and reduced expression of crucial proteins for heart function (connexin 43, tubulin, and dynein), as well as VCAM-1 and altered the cytokine expression profile in the heart. Finally, both EVs-inoculated and ICs-inoculated mice showed an increased presence of B-type natriuretic peptide (BNP) in serum, suggesting that EVs or ICs may participate in the onset of cardiac damage.

**Conclusions:**

Our results confirm the ability of EVs shed by the infective forms of *T. cruzi* and the immune complexes they form with IgG to induce cardiac alterations in mice similar to those described in the literature, in *T. cruzi*-infected mice as well as in Chagas disease patients. This study highlights the role of EVs in the pathogenicity of Chagas disease and reinforces the importance of considering them as virulence factors in the development of Chagas disease.

## Author summary

The World Health Organization considers Chagas disease one of the most important neglected diseases in the Americas. However, numerous Chagas disease patients are currently found worldwide, even in regions where the biological cycle of *T. cruzi* does not occur due to the absence of triatomines, which are essential for the parasite’s natural transmission (e.g., Europe, Australia, Asia, Canada, and the USA). The main pathology in the chronic phase of Chagas disease occurs at the cardiac level. In some cases, this is explained by the presence of intracellular amastigote nests in heart cells, while in others, cardiac manifestations appear despite the absence of the parasite in this organ. This has led several research groups to investigate the mechanisms responsible for cardiac damage in patients, which remain unclear. However, several hypotheses have been proposed: The first suggests that inflammatory reactions induced by the parasite itself or by any of its components, which cause damage to tissues, lead to tissue alterations. The second is based on molecular mimicry, resulting in autoantigen recognition by autoantibodies and an autoimmune attack. The third proposes the existence of factors capable of altering the conductivity of the heart’s conduction bundles, ultimately affecting cardiac contractility. Therefore, given the need to shed light on these mechanisms and based on the described protein load carried by these EVs and the physiological changes they are capable of inducing in uninfected cells, we have assessed the role of extracellular vesicles (EVs) shed by trypomastigotes—and the immune complexes they form upon recognition by host IgGs—in the development of cardiac alterations that could be associated with those described in Chagas disease patients. We believe that this work highlights the impact of EVs and immune complexes on the progression of Chagas disease symptoms.

## Introduction

Chagas disease (CD) is a parasitic infection caused by the protozoan *Trypanosoma cruzi*. This disease is a significant health problem, particularly in Latin America, where it is endemic. Currently, as a result of migratory flows to non-endemic areas, it is becoming a global health issue, i.e., 200,000–300,000 reported cases in the United States and more than 100,000 cases in Europe [1]. In Spain, where migration from endemic countries is common, it is estimated that there are at least 55,000 people infected in the chronic phase of the disease [2]. CD has been identified by the Centers for Disease Control and Prevention (CDC) and the World Health Organization as a neglected tropical disease [3–5].

CD is a zoonosis that affects humans as well as domestic and wild mammals. In endemic areas, *T. cruzi* infection is transmitted by hematophagous insects belonging to the Triatominae subfamily (Reduviidae family). The insect ingests blood containing trypomastigote forms, which differentiate into non-infective epimastigote forms in the insect’s midgut and begin to replicate. The parasites then migrate to the hindgut, where they differentiate into metacyclic trypomastigote forms that are excreted in feces, contaminating the skin or mucous membranes of another mammalian host. Metacyclic trypomastigotes can invade any nucleated cell in the mammal, transform into amastigotes, and, after replicating in the cytoplasm, parasites differentiate into trypomastigotes. Then, trypomastigotes induce host cell lysis and emerge in the blood and lymphatic streams to invade new cells or be ingested by another triatomine completing the cycle.

Other forms of transmission can occur, such as oral infection through the ingestion of contaminated food [6], transmission through blood transfusion or organ transplants from infected individuals [7,8] and congenital transmission by vertical transmission from mother to fetus [9,10]. The latter two routes are of epidemiological importance in geographical areas where there is no possibility of vector-borne transmission.

CD progression is divided into two phases: an acute phase and a chronic phase. The acute phase is usually asymptomatic, with patients exhibiting high blood parasitemia [11]. In some cases, patients might suffer mild cardiac anomalies such as sinus tachycardia and PR/QT prolongation, a diminution of voltage in QRS complexes and repolarization abnormalities in the electrocardiogram (ECG). Approximately 10% of infected individuals may present with clinical manifestations ranging from nonspecific symptoms to fulminant lethal myocarditis or infectious shock [12]. Patients presenting more severe electrocardiographic changes, including right bundle-branch block (RBBB), atrial fibrillation, or ventricular arrhythmias, have a worse prognosis [13]. Moreover, some immunocompromised patients may develop a fulminant acute disease, suffering an acute myocarditis, pericardial effusion, meningoencephalitis, and consequently death [14,15].

At the end of the acute phase, blood parasitemia is diminished by the activation of the immune system in most patients, but the immune response induced is not capable of resolving the infection, resulting in chronically infected patients. In the chronic phase of the disease, 30% of the patients present critical health concerns, including cardiac and digestive involvement, with cardiomyopathies being the most severe and frequent manifestation of the disease [16]. Chronic Chagas cardiomyopathy (CCC) is an inflammatory cardiomyopathy that can progress to dilated cardiomyopathy with heart failure, ventricular arrhythmias, conduction disturbances, stroke, and other systemic or pulmonary embolisms. Dilated Chagas cardiomyopathy refers to the hemodynamic pattern of CCC, characterized by left ventricular (LV) enlargement with global or segmental systolic function impairment, regardless of ECG findings. Several studies describe that heart failure due to CCC has a worse prognosis when compared to other cardiomyopathies [17,18].

Pathological manifestations of CCC result from abnormalities in electrical conductivity, myocardial contractile dysfunction, arrhythmias, or thromboembolism [19]. In advanced CD, the appearance of a significant LV dilation accompanied by a depression of global LV systolic function, which is directly correlated to the risk of death [20], has also been described. Although there is no specific early serum biomarker used to predict CCC, an elevation of B-type natriuretic peptide (BNP) that precedes advanced heart disease [21] has been described. In fact, in 2002, Ribeiro et al. reported an increase in BNP serum levels in patients and infected mice presenting LV systolic dysfunction and diastolic dysfunction associated with CD [22].

The mechanism by which *T. cruzi* induces cardiac pathology is still unclear, but several hypotheses have been proposed. One theory is based on inflammatory reactions induced by the parasite itself leading to tissue alterations. The second is based on molecular mimicry, which suggests an autoreactive process resulting from an alteration of the immune response [23–25] and nervous functionality [26,27], leading to the appearance of autoantibodies that may cause denervation in various organs, particularly the heart and digestive system, contributing to the dilations observed in the chronic phase of the disease and giving rise to the appearance of the different pathological manifestations of the disease. Finally, the last hypothesis is based on the persistence of the parasite in niches within the tissues [28,29]. These hypotheses may not be exclusive, and CCC may be the result of the combination of the different proposed mechanisms. During the infection, CD patients trigger an autoimmune response, recognizing numerous self-antigens such as actin and myosin, tropomyosin, laminin, galectin-1 and adrenergic receptors [30–36] and activation of T and B lymphocytes, which may contribute to the pathology [37,38]. It has been described how CD patients with cardiac involvement present elevated levels of inflammatory interleukins such as IFN-γ, TNF-α, and IL-6 and lower levels of regulatory interleukins such as IL-4 and IL-10 than those in the indeterminate phase [36,39]. Both Th1 and Th17 responses are protective against *T. cruzi* infection in the acute phase, but when they are maintained during the chronic phase, these responses may also contribute to the severity of cardiac damage [40,41].

Extracellular vesicles (EVs) are small membrane-coated vesicles released by nearly all cell types, including forms of *T. cruzi* [42]. They are classified based on size and composition into: a) exosomes (20–100 nm), b) ectosomes (100–1000 nm), and c) apoptotic bodies (>1000 nm) [43]. Nevertheless, currently other types of vesicles of larger sizes have been described, such as oncosomes and migrasomes [44]. The content of EVs includes proteins, lipids, and nucleic acids (DNA and RNA) [45]. EVs are known to play significant roles in the pathogenesis of CD, particularly in cell-cell communication, cell infection, and immune evasion [46,47]. In particular, EVs shed by infective forms of *T. cruzi* contain proteins considered as virulence factors such as phospholipase A2, GP63, cruzipain, and trans-sialidases, which make up 22% of their proteome, enzymes that catalyze the transfer of sialic acid from a glycoconjugate to an acceptor in the host cell, and which play a crucial role in the interaction and infection of *Trypanosoma cruzi*. and are involved in intracellular calcium mobilization [48,49].

Among cardiac diseases, including conditions such as rheumatoid arthritis, the role of the immune system in their etiology is gaining increasing recognition [50,51]. This involvement may be due to the presence of autoantibodies, as observed in lupus [52] or to an infectious etiology—such as bacterial or viral infections—where immune complexes formed by the patient’s immunoglobulins and pathogen antigens play a central role in cardiac pathology [53,54]. These immune complexes have been associated with myocardial cell damage and dysfunction, primarily through the activation of the complement system [55,56].

The presence of *T. cruzi* EVs was detected in the serum of chronic CD patients, forming immune complexes (EVs-IgG) with parasite-specific antibodies [57,58]. Both nuclear DNA and kinetoplast DNA of parasitic origin have been identified in EVs or immune complexes in the serum of CD patients, which may aid in confirmatory diagnostic tests [59]. The EVs released by trypomastigotes enhance parasitism, as shown *in vivo* in the hearts of mice inoculated with EVs and later infected with the parasite infective forms [60]. They also demonstrate immunomodulatory effects *in vitro*, including complement inactivation [57,61] and alterations in cytokine expression, similar to changes observed in the acute as well as chronic phases of CD in humans and animal models [27,62–68].

Recent studies showed that while trypomastigote EVs play an immunomodulatory role, immune complexes elicit varying responses in host macrophages based on whether the IgGs bound to EVs are sialylated [69]. These EVs also induce cellular alterations, such as increased cytosolic calcium, cell permeabilization, F-actin depolymerization, and disruption of the cell cycle [70]. Furthermore, interactions with non-infected cells lead to significant changes in mRNA expression, affecting 322 genes (168 overexpressed and 154 downregulated), particularly those involved in protein SUMOylation, signaling, and apoptosis [71].

In the present study, we analyzed the cardiac alterations induced in mice following intravenous inoculation of EVs obtained from bloodstream trypomastigote forms of *T. cruzi* and *in vitro*-formed immune complexes with anti-*T. cruzi* IgGs (EVs-IgG). We examined whether these EVs inoculations could induce autoantibodies in the inoculate mice and cause alterations in the electrocardiogram (ECG). Although no autoantibodies or evidence of infiltrates in the cardiac tissue were detected at 21 days, the inoculated mice exhibited ECG alterations, increased expression of inflammatory cytokines in cardiac tissue, elevated serum levels of B-type natriuretic peptide (BNP), dilation of the cardiac cavities, and decreased expression of connexin 43 and VCAM-1 among other proteins in myocardial tissue.

## Materials and methods

### Animal handling and authorization by the ethics and animal welfare committee

The use and handling of the animals in this study was carried out in accordance with institutional guidelines (Regulation of the Government of Spain: Royal Decree 53/2013) and European Union regulations (European Directive 2010/63/EU). The experimental design, the number of animals and the groups used in the study were studied and approved by the Ethics Committee of the University of Granada (Animal Experimentation Ethics Committee, 235-CEEA-OH-2018), as well as by the authorities of the Junta de Andalucía with the number 12/11/2017/162.

### Cell culture and parasite strain

Vero cells (ECACC 84113001) were cultured in 75 cm^2^ surface area culture flasks (Thermo Fisher Scientific, Waltham, MA, USA) using Minimum Essential Medium (MEM) (Sigma Aldrich, St. Louis, MO, USA) supplemented with 10% heat-inactivated fetal bovine serum (iFBS) (Gibco, Waltham, MA, USA) plus antibiotics (penicillin 100 U/mL; streptomycin 100 μg/mL). The cell cultures were maintained at 37 °C, in a moist atmosphere enriched with 5% CO2.

Vero cells were initially infected with purified trypomastigotes obtained *in vitro* from *T. cruzi* strain Pan4 (Tc Ia + Tc Id), as previously described by Diaz Lozano [57]. After 120 h of intracellular development of the parasite, the trypomastigotes present in the cell culture supernatant were harvested, washed and isolated by successive centrifugation processes.

Experiments involving manipulations with the infective forms of the parasites were conducted in the NCB3, biological containment laboratories (A/ES/23/I-40) at the University of Granada.

### Obtention of EVs secreted by trypomastigotes

To obtain EVs, the methodology previously described by Díaz Lozano et al. was followed [57]. The purified cell derived trypomastigotes forms were incubated for 5 h at 37°C in MEM medium (Sigma Aldrich, St. Louis, MO, USA) without iFBS. After incubation, trypomastigote viability was assessed by trypan blue staining, showing a percentage close to 95% of live forms. The parasites were then removed by centrifugation at 1000 xg for 15 min and the supernatant was collected and centrifuged at 17,000 xg for 30 min at 4°C in order to remove cell debris and apoptotic blebs. This supernatant was filtered through a 0.22 μm pore filter (Sartorius, Göttingen, Germany) and ultracentrifuged at 100,000 xg for 4 h to obtain the EVs. These ultracentrifugation steps were performed in a CP100NX ultracentrifuge (Hitachi Koki, Tokyo, Japan) with a P70AT fixed-angle rotor. The resulting pellet containing the EVs was washed three times by ultracentrifugation, using 0.1 µm-pore filtered sterile PBS. Finally, the EVs were concentrated by ultracentrifugation using a P50A3 fixed-angle rotor and resuspended in 100 µL of sterile filtered PBS.

The hydrodynamic size distribution of the purified EVs was measured by Nanoparticle Tracking Analysis (NTA), using a Nanosight instrument equipped with a sample chamber, a 405-nm laser and a high-sensitivity complementary metal-oxide-semiconductor (CMOS) camera; size analysis was performed using NTA 2.3 image analysis software (NanoSight Ltd., Amesbury, UK). The isolation procedure was also evaluated by transmission electron microscopy (TEM). Briefly, samples obtained after the final ultracentrifugation purification step were resuspended in 30 µL of Tris-HCl (pH 7.3), and 5 µL of the suspension was placed directly onto Formvar/carbon-coated grids. After allowing the sample to adsorb onto the grid for 30 minutes, the grids were washed with PBS and fixed in 1% glutaraldehyde for 30 minutes. They were then washed with PBS and stained with 2% (v/v) uranyl acetate. Finally, the samples were observed under a Carl Zeiss SMT LIBRA 120 PLUS TEM microscope (Carl Zeiss, Oberkochen, Germany). The size of the nanoparticles was measured using the microscope’s own measurement scale and ImageJ 1.41 software to calculate the mean and mode sizes.

Protein quantification of the EVs samples was performed using the Micro-BCA Protein Assay Kit (Thermo Fisher Scientific, Waltham, MA, USA) following the manufacturer’s protocol.

### Production of polyclonal anti-*T. cruzi* antibodies and immune complex (EVs-IgG) formation

The methodology previously described by Cornet Gomez et al. was followed [71]. Briefly, for mouse immunization, three 6-week-old female CD1 mice were injected intramuscularly once a week for six weeks.

For the preparation of a total extract of *T. cruzi* trypomastigotes from the Pan4 strain, 10^9^ trypomastigotes were isolated from cell culture. The trypomastigote forms underwent three cycles of freezing at -20 °C and slow thawing at 4 °C, followed by sonication in a Branson SLP device (10 s on, 10 s off, for 2 min).

The trypomastigote extract, combined with an adjuvant, was injected (10 μg of protein extract per dose) using a suspension of whole parasite extract in PBS plus Freund’s complete adjuvant (Sigma Aldrich, St. Louis, MO, USA) in a 1:1 ratio. Prior to the first immunization, a blood sample was collected to obtain pre immune control serum. Freund’s complete adjuvant was used only for the initial immunization, while incomplete Freund’s adjuvant (Sigma Aldrich, St. Louis, MO, USA) was used for subsequent injections.

Blood samples were obtained weekly from the submandibular vein after the first two immunizations, and antibody titters were assessed by indirect ELISA in 96-well plates (Maxisorp, Thermo Fisher Scientific, Waltham, MA, USA) coated with 5 μg of the trypomastigote lysate per well in carbonate buffer (0.1 M sodium carbonate, 0.1 M sodium bicarbonate, pH 9.4). At the end of the immunization period, the mice were euthanized via intraperitoneal injection of sodium pentobarbital (Labiana Life Sciences S.A., Barcelona, Spain), following ethical guidelines. Blood samples were then collected by cardiac puncture, and sera with titters higher than 1:6400 were obtained using BD Microtainers and the serum stored at −80 °C (BD, Franklin Lakes, New Jersey, USA). The anti-*T. cruzi* IgGs from mice were purified from sera using a Protein G High Performance Spintrap (Cytiva, Washington, D.C., USA) following the manufacturer’s instructions.

The formation of the immune complexes has already been described by [69]. Briefly, to carry out the formation of immune complexes *in vitro*, equal concentrations of EVs protein were incubated with anti-*T. cruzi* IgGs for 90 minutes at 37^o^C under gentle agitation. Thereafter, the immune complexes were ultracentrifuged at 100,000 xg for 4 hours at 4 °C to remove the supernatant containing unbound immunoglobulins, and the pellet was washed three times by ultracentrifugation at the same speed in sterile PBS filtered through 0.22 μm pore filters. The protein concentration in the supernatant after ultracentrifugation was assessed to ensure that no proteins remained, confirming the complete removal of unbound IgG.

### EVs and Immune Complexes Inoculation. Electrocardiograph analysis

The experimental design including the sample size per group, the timing of interventions and experiments conducted, was designated in collaboration with the Animal Welfare Committee of the Animal Ethics Committee at the University of Granada (UGR) for the management of experimental animals is shown in S1 Fig. The samples were intravenously injected through the marginal tail vein into three groups of five adult female CD1 strain mice. The average weight of the mice used was 26.34 g, which corresponds to an estimated blood volume of approximately 7% of their body weight (about 1.84 mL) [72]. One group was injected with EVs (equivalent to 1.08 µg/mL of EV protein per mL of blood in inoculated mice, corresponding to approximately EVs 2.8 x 10^7^ EVs, per inoculation and mouse). A second group was inoculated with immune complexes (formed by the same amount of EV proteins and an equivalent amount of purified IgG, formed as described above. The third group received sterile PBS, used as the vehicle for EV or immune complex suspension, serving as a negative control as it is not expected to induce immunological stimulation. The inoculated volume was 50 μL per inoculation and mouse. The EV dosage was based (i) the effective dose 50 (ED₅₀) reported for *T. cruzi* EV effects on host cells [70]; (ii) the levels of circulating EVs in the serum of Chagas disease patients [57] and (iii) available literature data regarding the half-life of inoculated EVs and the number of EVs circulating in plasma, as further detailed in the Discussion section.

Before the first sample inoculation, an ECG was performed on each mouse using a Physiological Monitoring System for Mice (Harvard Apparatus, Holliston, MA, USA); these measurements were used as the cardiac baseline. Prior to the electrocardiogram measurement, the mice were anesthetized with 1.5% isoflurane atmosphere for three minutes. Electrodes were placed on the animal extremities, and after a 90 second adaptation period, the measurements were recorded for 30 seconds.

Mice were inoculated twice a week for three weeks, with ECGs performed 30 minutes post-inoculation. The electrocardiogram analysis included measuring the heart rate of the mice and examining the QRST wave (P1, P2, Q, R, S, T, and Tfinal). A total of 20 QRST waves were analyzed for each mouse and measurement, using the LabChart Reader software (ADInstruments, Oxford, UK). After the final ECG, blood samples were collected from the submandibular vein of each mouse. Finally, the mice were euthanized by intraperitoneal injection of sodium pentobarbital (Labiana Life Sciences S.A., Barcelona, Spain).

### Anti-EV, anti-*T. cruzi* antibodies and cardiac autoantibodies presence in the sera of injected mice

The levels of anti-EVs antibodies generated in the mice after 21-days inoculation were determined by indirect ELISA using as antigen a EVs extract obtained after its lysis with RIPA buffer (50 mM Tris HCl, 150 mM NaCl, 1.0% IGEPAL, 0.5% Sodium Deoxycholate, 1.0 mM EDTA, 0.1% SDS, pH of 7.4). A lysate of trypomastigote forms was used as a control. For this purpose, 96 wells plate (Maxisorp, Thermo Fisher Scientific, Waltham, MA, USA) were sensitized overnight at 4°C with 2 μg of EVs lysate or 5 μg trypomastigote lysate per well in carbonate buffer (0.1 M sodium carbonate, 0.1 M sodium bicarbonate, pH 9.4). After sensitization, the plate was washed three times with washing buffer (PBST, PBS + 0.1% Tween 20) and subsequently blocked with blocking buffer (PBST + 5% non-fat milk) overnight at 4°C. Sera from the different mice were diluted 1:200 and incubated for 120 minutes at RT. After incubation, the plate was washed five times with PBST, and HRP-conjugated secondary anti-mouse immunoglobulins (Agilent, Santa Clara, CA, USA) was added and incubated for 60 minutes at RT. After five additional washes, peroxidase substrate solution was added and incubated for 20 minutes in the dark. The reaction was stopped by adding 3 M HCl, and the absorbance was measured at 492 nm using a Multiskan Spectrum Reader (Thermo Fisher Scientific, Waltham, MA, USA).

The presence of cardiac autoantibodies was measured by indirect ELISA. For this purpose, a control mouse heart was washed in PBS to remove blood residues, the heart was then cut into 3 mm^3^ pieces and placed in tubes containing 2.8 mm ceramic beads (OMNI International, Kennesaw, GA, USA). The tubes were subjected to three 30-second shaking cycles in a Precellys 24 tissue homogenizer at 4°C (Bertin Technologies, Paris, France). The heart extract was then sonicated with ten 10-second pulses and centrifuged at 16000 × g at 4°C. The supernatant, containing the proteins, was transferred to a new tube, and the protein concentration was measured using the Micro-BCA Protein Assay Kit (Thermo Fisher Scientific, Waltham, MA, USA), following the manufacturer’s protocol.

The presence of autoantibodies specific to the β1-adrenergic receptor in cardiac tissue was also assessed by indirect ELISA. To this end, a synthetic peptide with the sequence GDRPRASGCLARAG, corresponding to the C-terminal intracellular domain of the receptor, was synthesized and used as an antigen (LifeTein, Hillsborough, New Jersey, USA).

The ELISA was performed as described above, but the plates were sensitized with 5 μg of heart extract or synthetic peptide, respectively. To assess antibody avidity and remove low-affinity antibodies, KSCN was used as a chaotropic agent to increase the assay’s stringency as described elsewhere [73]. Briefly, after primary antibody incubation and three washes with PBS-T, half of the wells were incubated with 0.5 M KSCN for 15 minutes at 37°C. The wells were then washed three times with PBS-T, followed by the subsequent steps described above. In this ELISA, an aliquot of the anti-*T. cruzi* serum, from which IgGs were isolated to form the ICs *in vitro*, was assessed to rule out recognition of the mouse heart.

### Brain natriuretic peptide (BNP)

To assess brain natriuretic peptide (BNP) levels in mouse serum as a marker of heart failure, the RayBio Mouse/Rat Brain Natriuretic Peptide EIA competitive ELISA kit (RayBiotech Life, Peachtree Corners, GA, USA) was used, following the manufacturer’s protocol. A microtiter plate was sensitized for 90 minutes at room temperature (RT) with 100 µL of anti-BNP antibody supplied with the kit. Then, four subsequent washes with washing buffer were carried out. Standard solutions of biotinylated BNP were prepared at 1,000 pg/mL, 100 pg/mL, 10 pg/mL, 1 pg/mL, 0.1 pg/mL, and 0 pg/mL to develop a standard curve. For peptide determination in serum samples, sera were diluted 1:4, and 10 pg/mL biotinylated BNP were added. Known concentrations of BNP and samples were added to the wells of the plate, which were incubated for 12 hours at 4°C. After plate incubation, four washes were performed, followed by incubation with streptavidin-HRP solution for 45 minutes at RT. Subsequently, the plates were washed four times with washing buffer and TMB One-Step substrate reagent was added and incubated for 30 minutes at RT in the dark. Thereafter, a stop solution was added, and the absorbance was read at 450 nm using a Multiskan Spectrum reader (Thermo Fisher Scientific, Waltham, MA, USA).

### Gene expression analysis by RT-qPCR

Samples of 30 mg of cardiac tissue from mice were placed in RNAlater, to preserve the RNA, in RNase-free tubes and homogenized prior to nucleic acid isolation. The samples were homogenized in 600 µL of lysis buffer from the RNA purification kit (RNeasy Mini, Qiagen, Hilden, Germany) along with 2.8 mm ceramic spheres (OMNI International, Kennesaw, GA, USA). The tubes were subjected to three 30-seconds shaking cycles in a Precellys 24 tissue homogenizer at 4°C (Bertin Technologies, Paris, France).

RNA purification was conducted with the RNeasy Mini kit (Qiagen, Hilden, Germany) following the manufacturer’s instructions. Elution was performed adding 40 µL of RNase-free water. The eluted nucleic acids were treated with DNase I RNase free (Thermo Fisher Scientific, Waltham, MA, USA), 5 µL of 10x buffer were added and 2 µL of DNase I and incubated at 37^o^C for 30 minutes. After this digestion time, 1 µL of 50 mM EDTA was added, and the enzyme was inactivated by heat incubating it at 65^o^C for 10 minutes. RNA was precipitated adding one volume of absolute ethanol, incubating it overnight at -20^o^C. RNA was isolated by centrifugation, washed with ethanol 70% and centrifuged again. The RNA pellet was resuspended in RNase-free water. and quantified with NanoDrop One (Thermo Fisher Scientific, Waltham, MA, USA). As a method to verify the absence of genomic DNA in the sample, a conventional PCR was performed using actin primers to check for contaminant DNA. The PCR showed no amplification for actin gene.

For RT-qPCR analysis iTaq Universal SYBR Green One-Step Kit (BioRad, Hercules, CA, USA) was employed, following the manufacturer’s instructions. Briefly, reactions were performed in 10 µL final volume using a CFX-96 Real Time System (BioRad, Hercules, CA, USA), for each reaction 300 nM of each primer and 100 ng of RNA were employed. The thermocycler program consisted of a first retrotranscription step at 50 °C for 10 minutes, followed by an enzyme activation and DNA denaturation step at 95 °C for 1 minute, followed by 40 cycles consisting of a denaturation step at 95 °C for 10 seconds and an annealing and extension step at 60 °C for 30 seconds and then a plate read. A final melt gradient step was carried out at the end of RT-qPCR reactions, ranging from 65 °C to 95 °C in 0.5 °C increments. *gapdh* was employed as the normalizer gene and protein expression was normalized to the PBS injected mice control. The expression of the following proteins was analyzed.: *IL-1β*, *IL-4*, *IL-6*, *IL-10*, *IL-12*, *IL-13*, *IL-15*, *IL-18*, *IL-23*, *IL-25*, *IFN*γ, *TNFα*, *TGFβ*, *G-CSF*, *Fas*, *FasL*, *N*OS, and *Cx43* (S1 Table).

### Histological analysis

For histopathological evaluation, freshly extracted hearts from each group were cut longitudinally and washed in PBS to remove most of the blood. They were immediately fixed in 10% neutral-buffered formalin for 24 hours to preserve the tissue. These preserved samples were embedded in paraffin wax, and 5 μm-thick sections were mounted on slides. Sections were dewaxed with xylol and rehydrated in decrescent concentrations of ethanol and stained with H&E and Picrosirius Red. Briefly, H&E staining was performed incubating the slides with Harris hematoxylin for 2 minutes, washed and incubated with alcoholic eosin for 45 seconds. Picrosirius Red staining was performed for fibrosis quantification. Images were acquired in an Axio Scope.A1 microscope (Carl Zeiss, Germany). The hematoxylin-stained preparations were used to observe the general structure of the cardiac tissue as well as to measure the areas of the heart chambers (left and right ventricles) and the thickness of the cardiac walls. These measurements were performed using ImageJ software, analyzing at least 8 slides from each mouse. The Picrosirius Red-stained preparations were observed under polarized light to visualize collagen fibers. The quantification of collagen was performed using ImageJ software, analyzing 12 slides from each mouse.

### Confocal microscopy

For immunohistochemical studies, the deparaffinized slides were permeabilized by incubating them in PBS-Triton X100 (0.1%). Subsequently, they were incubated with an antigen unmasking solution (10 mM sodium citrate, pH 6) and placed in a pressure cooker for 2 minutes, followed by three washing steps with a washing buffer (PBST). The samples were blocked using a blocking buffer (PBST + 1% OVA) for 30 minutes. After three washing steps, the slides were incubated with the different primary antibodies for 90 minutes in a humid dark chamber. The antibodies used were: anti-VCAM1, dilution 1:10 (Invitrogen, Waltham, MA, USA), anti-VLA-4, dilution 1:1000 (Invitrogen, Waltham, MA, USA), anti-dinein, dilution 1:500 (Invitrogen, Waltham, MA, USA), anti-tubulin, dilution 1:200 (Cytoskeleton, Denver, CO, USA), anti-connexin 43, dilution 1:400 (Sigma Aldrich, St. Louis, MO, USA). After primary antibody incubation, five washing steps were performed, secondary antibodies employed were anti-rat IgG-alexa fluor 633, diluted 1:500 (Thermo Fisher Scientific, Waltham, MA, USA), anti-rabbit IgG-alexa fluor 647, diluted 1:500 (Thermo Fisher Scientific, Waltham, MA, USA), anti-sheep IgG-alexa fluor 633, diluted 1:500 (Thermo Fisher Scientific, Waltham, MA, USA) and anti- rabbit IgG-FITC, diluted 1:80 (Sigma Aldrich, St. Louis, MO, USA). After secondary antibody incubation, 5 washing steps were performed, and 25 μL of VECTASHIELD Antifade Mounting Medium with DAPI (Vector Laboratories, Newark, CA, USA) were placed over the slides and covered with a cover slip for confocal microscopy. Samples were examined under a Leica DMI6000 confocal laser microscope equipped with an argon laser of 496 nm and a He/Ne laser of 633 nm. Fluorescence intensity was measured with Image J software analysing at least five images of each group of animals.

### Statistical analysis

Statistical analyses were performed using one-way and two-way analysis of variance (ANOVA) to evaluate the differences among experimental groups. The one-way ANOVA was applied to compare a single factor across multiple groups, while the two-way ANOVA was used to assess the effects of two independent variables and their interaction. Post hoc multiple comparisons were conducted using the Bonferroni test to identify specific group differences. Results are presented as mean ± SEM, and statistical significance was defined as p < 0.05. All analyses were performed using GraphPad Prism version 9.

## Results

### Characterization of the EVs obtained

The purity and size of the EVs were assessed by TEM, their diameters were quantified using Image J (S2A and S2B Fig). The results of the size distribution study by nanoparticle tracking analysis (NTA) showed a population of EVs with a mean size of 122.8 +/− 1.3 nm and a mode of 91.7 +/− 0.6 nm (S2C Fig). According to the literature, this range of sizes corresponds to exosomes.

The recognition by antibodies obtained from the mouse immune serum against the total antigens of the EVs shed by *T. cruzi* is shown in S2D Fig The proper formation of the immune complexes was previously evidenced by atomic force microscopy, where changes in the profile were observed as a result of the binding of the anti-*T. cruzi* antibodies to the EVs shed by trypomastigotes [69].

### EVs or immune complexes inoculation induces ECG changes in mice

**Fig 1:**
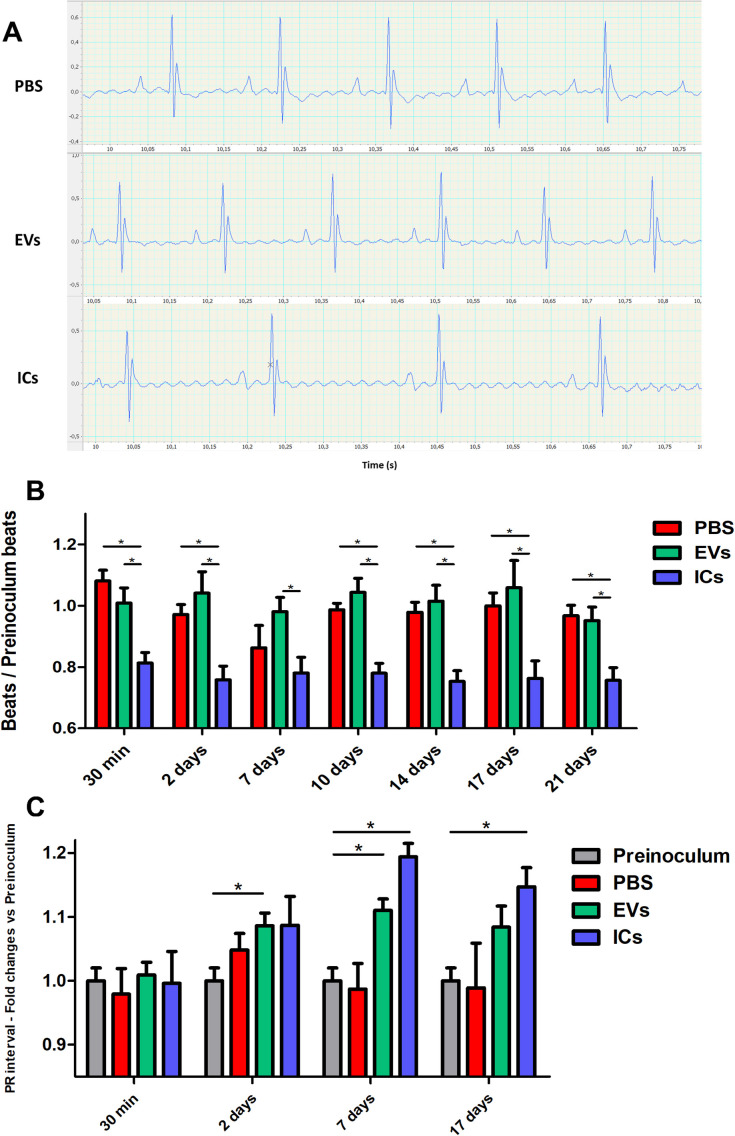
Effects of EVs (green) or immune complexes (blue) on mice cardiac activity. **(A)** Representative ECG registries of controls (PBS) and animals stimulated with EVs or ICs at different timepoints along the experiments. **(B)** Cardiac frequency in beats per minute of experimental mice/beats per minute of preinoculated mice; ICs-stimulated mice presented bradycardia. **(C)** PR interval of the ECG; Both EVs and ICs stimulation increased the PR interval in mice. The values represent the mean ± SEM, with significance set at p < 0.05(*).

The global records obtained from the electrocardiographic analysis are shown in Fig 1, depicting the variations in heart rate throughout the different intravenous inoculation of the experiment showed that immune complexes produced a decrease in the beats per minute compared to controls, while EVs led to a non-statistically significant increase (Fig 1A). This effect was observed after the first inoculation and persisted throughout the entire experiment (Fig 1B). These results suggest an involvement of cardiac conduction, specifically in the sinoatrial node, or bradycardia due to an alteration of the autonomic nervous system, with parasympathetic predominance. Bradycardia may predispose to heart failure due to reduced cardiac output and cavity dilation.

PP, QRS and QT intervals were measured based on the ECG results, but no statistically significant differences were found between the different groups. QTc measurements showed an early trend towards an increase in QTc interval in EVs and ICs, which did not persist over time (S2 Table). The group of mice inoculated with EVs (at days 2 and 7) or immune complexes (at days 7 and 17) showed an increase in the PR interval (Fig 1C).

### Humoral response induced after intravenous inoculations of EVs or ICs (IgG-EVs)

**Fig 2:**
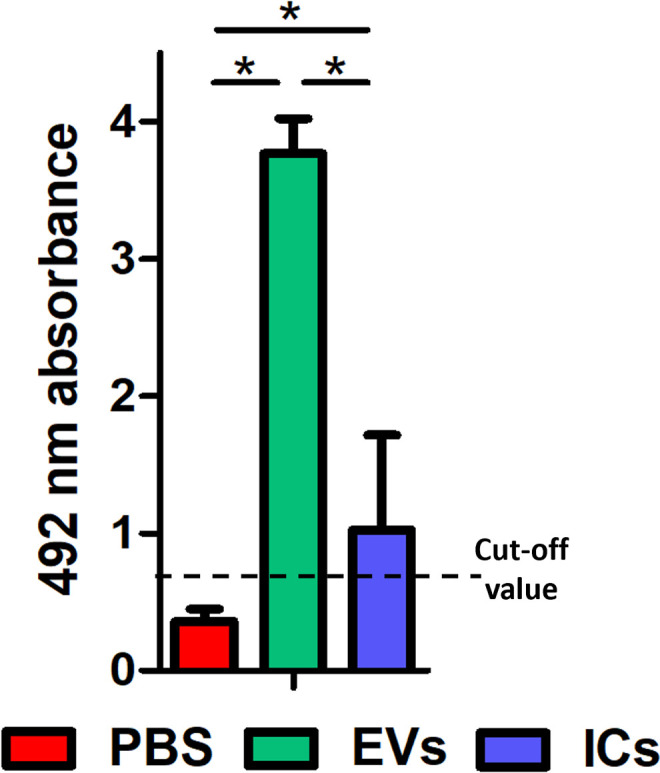
Humoral immune response observed in mice after the inoculation with EVs (green) or ICs (blue). Higher levels of specific antibodies against EVs were detected in mice receiving EVs compared to mice injected with the ICs. The values represent the mean ± SEM, with significance set at p < 0.05(*). Cut-off value = Mean of the PBS-injected mice absorbance + 3xSD.

The ability of mice to generate an antibody response against EVs following 21 days of intravenous inoculation with EVs and ICs is shown in Fig 2, demonstrating their capacity to produce specific antibodies against the antigens present in the parasite’s EVs. In both groups, absorbance levels were higher than the cut-off determined by the serum of control animals inoculated with PBS. Although both inocula contained the same amount of antigen, the inoculation of free EVs exhibited a greater antigenic capacity than that of immune complexes, as reflected in the average absorbance values in the graph. As a control, the recognition of whole *T. cruzi* trypomastigote lysate antigens was evaluated using different mice sera, showing similar results (S3 Fig).

The assessment of the generation of autoantibodies after 21 days of inoculation of parasite EVs or ICs was carried out by an indirect ELISA against a mouse heart extract and against a synthetic peptide corresponding to the β1-adrenergic receptor. This analysis revealed that neither EV-injected nor IC-injected mice developed autoantibodies recognizing cardiac tissue or the β1-adrenergic receptor, not even those with low-affinity binding (S4 Fig). Similarly, the anti-*T. cruzi* IgGs used to form the ICs *in vitro* did not exhibit cardiac recognition (S4 Fig).

### Heart alterations were induced by EVs and ICs injection in mice

**Fig 3:**
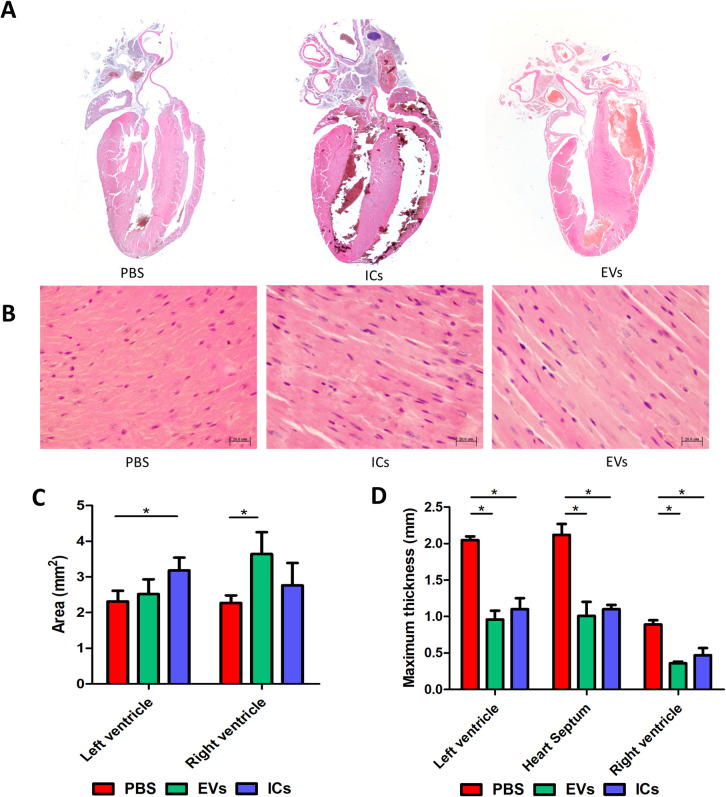
Heart morphological alterations induced by EVs (green) and ICs (blue). **(A,B)** Histological analysis of the cardiac longitudinal sections showed no relevant alterations or significant immune infiltration. **(C,D)** Measurement of H&E-stained mouse heart sections revealed an increase in the mean area of the right and left ventricles produced by the administration of EVs and ICs, respectively **(C)** Both EVs and ICs induce wall thinning in both ventricles as well as in the heart septum **(D)**. The values represent the mean ± SEM, with significance set at p < 0.05 (*).

After completion of the experiment and euthanasia of the animals (21 days after the first inoculation and seven injections as described in the methodology), samples of the hearts of mice from the three experimental groups were analysed histologically, stained with H&E. Morphological analysis of longitudinal sections of the heart did not demonstrate the presence of relevant lesions in the myocardium or valves, nor significant leukocyte infiltration. None of the groups showed phenomena of myocarditis, pericarditis or vasculitis under optical microscopy (Fig 3A and 3B).

However, measurements of the areas of the left and right ventricles showed a statistically significant dilation of the left ventricle in mice injected with immune complexes compared with negative controls (3.18 ± 1.15 mm^2^ vs. 2.31 ± 0.96 mm^2^), whereas mice injected with EVs showed dilation in the right ventricle (3.64 ± 1.55 mm^2^ vs. 2.27 ± 0.67 mm^2^) (Fig 3A and 3B).

Both groups showed a reduction in the thickness of the walls of the left and right ventricles, as well as the cardiac septum. Cardiac wall thinning was more pronounced in mice injected with EVs than in those inoculated with ICs (Fig 3A and 3C).

**Fig 4:**
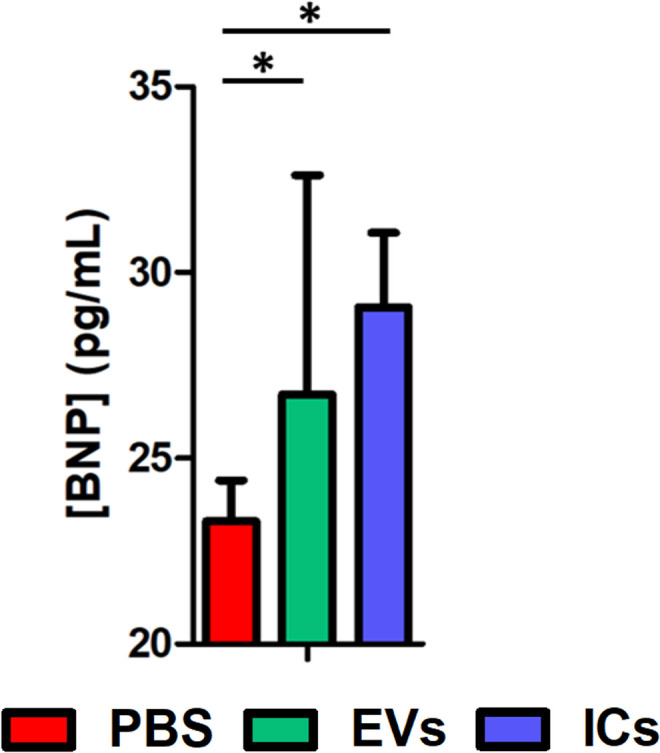
BNP serum levels obtained after inoculation of animals with EVs (green) and ICs (blue) when compared to the negative control (red). The values represent the mean ± SEM, with significance set at p < 0.05 (*).

After observing the dilation of the cardiac cavities, a physiopathological test was conducted to assess the serum levels of circulating BNP in treated animals compared to control mice inoculated with PBS. To this end, brain natriuretic peptide (BNP) levels were analyzed in the sera of animals following EV or IC injection. The analysis revealed an increase in BNP concentration compared to control mice. Mice injected with EVs showed a concentration of 26.72 ± 5.91 pg/mL, and mice injected with ICs presented a concentration of 29.07 ± 2.00 pg/mL, while sera from control mice showed a concentration of 23.32 ± 1.11 pg/mL. These data confirm the presence of signs of cardiac damage under the studied conditions (Fig 4).

### Confocal microscopy analysis revealed decreased synthesis of structural cardiac proteins

**Fig 5:**
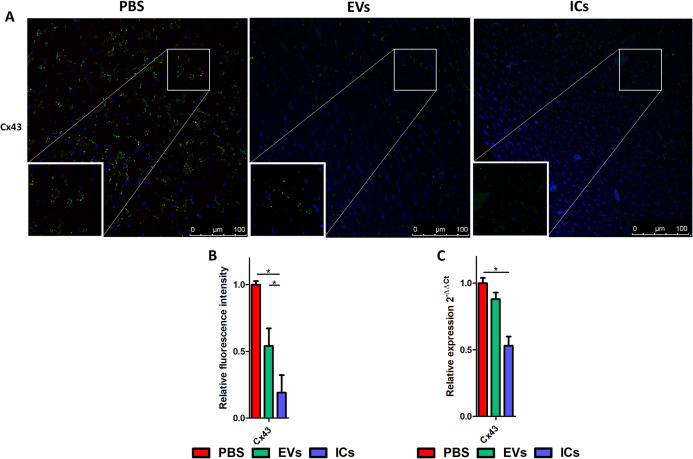
Analysis of connexin 43 expression in mice inoculated intravenously with EVs (green) or ICs (blue). Confocal microscopy revealed decreased connexin 43 synthesis in mice injected with EVs and ICs **(A)**. Fluorescence intensity was quantified using ImageJ, with values normalized to controls. Slides were stained with DAPI (nuclei in blue), and connexin43 was detected using monoclonal antibodies followed by labeling with a FITC-conjugated secondary antibody. **(B)**. *connexin 43* expression levels in mice injected with EVs or EVs-ICs were further assessed by RT-qPCR **(C)**. The values represent the mean ± SEM, with significance set at p < 0.05 (*).

Analysis of connexin 43 protein presence in heart sections by confocal microscopy, using anti-connexin 43 as the primary antibody followed by fluorescence intensity analysis with ImageJ, revealed a statistically significant decrease in protein presence in mice inoculated with EVs. This decrease was even more pronounced when they were inoculated intravenously with ICs (Fig 5A and 5B). These results were confirmed by RT-qPCR, as shown in Fig 5C.

**Fig 6:**
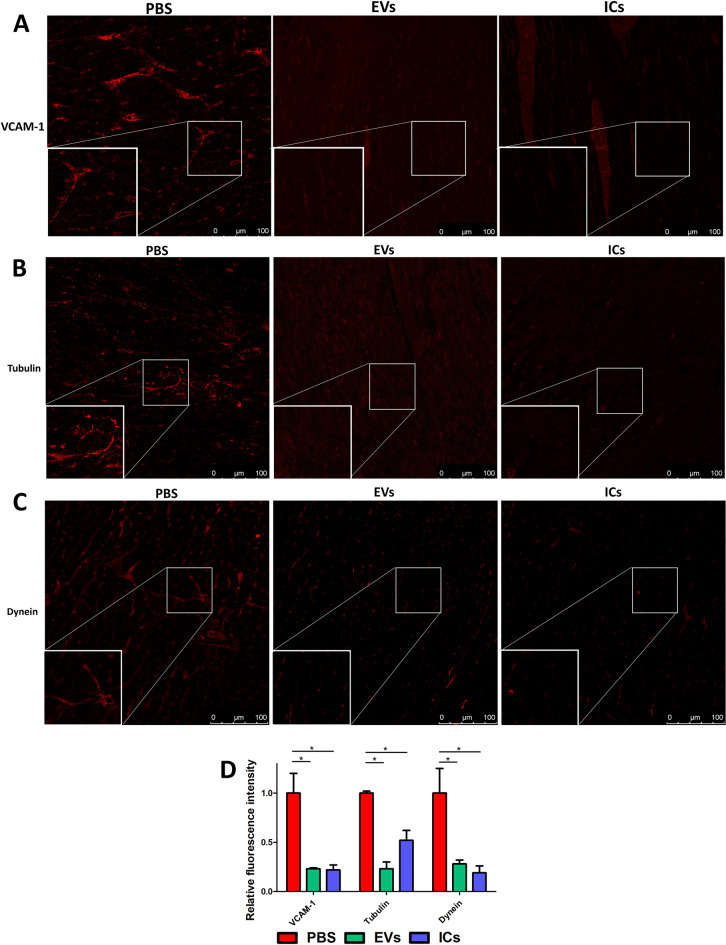
Decreased synthesis of VCAM-1, tubulin and dynein proteins in the heart of mice stimulated with EVs (green) or ICs (blue). Confocal microscopy images of heart sections incubated with specific antibodies against VCAM-1 **(A)**, tubulin **(B)** and dynein **(C)** showed a decrease in fluorescence signals in samples from the EVs and ICs groups. Fluorescence intensity was quantified by ImageJ, and values referred to the intensity present in controls (PBS) **(D)**. The values represent the mean ± SEM, with significance set at p < 0.05 (*).

Confocal microscopy analysis also showed decreased synthesis of VCAM-1, a key protein in the vascular endothelium that mediates lymphoid cell adhesion and facilitates the extravasation of lymphoid cells into tissues. In both groups of injected mice, the presence of this protein in the cardiac tissue was lower than in control mice (Fig 6A and 6D). This fact is consistent with the absence of infiltrates observed histologically (Fig 3) and with the absence of collagen deposition, studied by Picrosirius red staining and subsequent observation using dipolarized light microscopy, which would correspond to an absence of fibrotic processes, after repeated inoculation of EVs or ICs (S5).

Other proteins involved in cardiac contractility, such as dynein and tubulin, were also analyzed and are shown in Fig 6. A decrease in their expression was observed in all experimental groups compared to the control hearts.

### EVs or ICs inoculation produced changes in the heart cytokine profile

**Fig 7:**
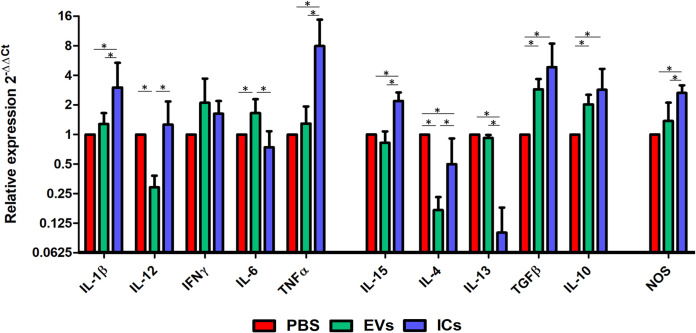
Relative expression of cytokines in the hearts of animals at the end of the experiment (21 days), following repeated inoculations with *T. cruzi* EVs (green) or ICs (blue). Cytokine expression was normalized to the expression of *gapdh* as a housekeeping gene and compared to the expression in control PBS-injected mice (red). The values represent the mean ± SEM, with significance set at p < 0.05 (*).

Fig 7 shows the results of the cytokine profile analysis in the heart tissue of mice inoculated with EVs or ICs. Mice stimulated with EVs (green) exhibited overexpression of *IL-6* and downregulation of *IL-12* and *IL-4*, whereas mice stimulated with ICs (blue) showed upregulation of *IL-1β*, *TNF-α*, *IL-15*, and *NOS*, along with downregulation of *IL-4* and *IL-13*. Both inoculations increased the expression of *TGF-β* and *IL-10* compared to expression values of control mice.

## Discussion

In this work, we aimed to analyze the influence of EVs released by infective trypomastigote forms of the Pan4 strain (DTU Iac) [74] on the development of autoantibodies that could induce cardiac alterations in mice following periodic intravenous injections of these EVs or immune complexes (EVs-IgG). Although we did not detect autoantibodies against cardiac tissue or the β1-adrenergic receptor, experimental mice mounted a humoral response, developing specific antibodies recognizing *T. cruzi* antigens, with higher levels in the EVs-injected group than in the ICs-injected mice. Furthermore, our results show that after the first intravenous inoculation of EVs or ICs, cardiac rhythm alterations were observed, suggesting a direct effect of EVs or ICs on cardiac rhythm compared to control mice injected with the same volume of PBS.

The initial aim of this study was to evaluate the capacity of EVs or IC to the production of autoantibodies similar to those found in patients with CD, which are believed to be contribute to cardiac pathology. This hypothesis is based on the protein and nucleic acid cargo of the EVs [49,75] which may potentially trigger an autoimmune response by generating antibodies that recognize shared epitopes between parasite-derived antigens and host components. Additionally, EVs might induce cellular damage via complement activation, a mechanism previously demonstrated in endocarditis of bacterial origin [56,76]. However, both the infectious forms of *T. cruzi* and the trypomastigotes derived EVs, have been shown to inhibit complement activation by interfering with C3 convertase activity [77,78]. Similarly, Diaz Lozano et al. 2017, showed that EVs from infectious forms of the parasite were capable of inhibiting complement activity and that this inhibition was related to the surface glycoproteins (MASP) of the parasite EVs [57]. Therefore, in our model, complement-mediated damage is unlikely to occur and was not considered a relevant mechanism.

Previous work has shown that EVs from *T. cruzi* trypomastigotes exert multiple effects on host cells [70], including: (i) increased intracellular calcium due to mobilization from internal stores such as the ER and mitochondria; (ii) disruption of the cytoskeleton, particularly actin and vimentin; (iii) permeabilization of host cells to macromolecules, including α-sarcin (18 kDa) and IgGs (150 kDa), potentially promoting vascular leakage; and (iv) enhancement of parasite infectivity *in vitro*. These effects could favor the emergence of autoantibodies. Furthermore, Retana Moreira et al. (2019) determined that these phenomena were specific to *T. cruzi*-derived EVs and correlated EV protein content with vesicle abundance—estimating that 5 × 10¹⁰ EVs (from PAN4 strain trypomastigotes) contain ~12 µg of protein. The effective dose 50 (ED₅₀) was calculated in terms of EV protein content required to induce cellular effects.

In parallel, Díaz Lozano et al. (2017) quantified parasite EVs or ICs in the serum of patients with chronic cardiac Chagas disease, finding concentrations around 37.2 µg/mL of EV protein [57]. Additionally, Auber & Svenningsen (2022) reported average concentrations of ~4.5 × 10¹⁰ EVs/mL in healthy human serum [79]. EV half-life in circulation—prior to uptake by phagocytes—has been estimated to range from 30 minutes to 5.5 hours, independent of their origin, based on studies in humans undergoing plasmapheresis [80–82].

Considering these data and the ED₅₀ values established for *T. cruzi* EVs on cultured cells [70], we extrapolated an effective dose (ED₉₀) to determine the optimal quantity of EVs for *in vivo* administration. This dose was designed to achieve maximal biological effect while staying within the physiological range of EV concentrations observed in CD patients. Importantly, the ICs used in our study were composed of mouse IgGs (from the same strain), and thus incapable of eliciting an immune response. The vehicle (PBS) used for suspending EVs and ICs was protein-free and physiologically balanced. Based on this rationale, the EV dosage was adjusted to ensure adequate exposure in the bloodstream to assess their potential to induce autoantibodies.

The previously described increase in the PR interval suggests an alteration in the conduction tissue, mainly in the atrioventricular node, which could indicate a first-degree atrioventricular block. This predisposes to asynchrony between atrial and ventricular contraction (electromechanical decoupling), which could lead to a reduction of cardiac output and exacerbating heart failure, similar to what has been described *in vivo* in animals or patients with Chagas cardiomyopathy. The described ECG alterations, such as bradycardia and an increased PR interval, clearly indicate a delay in electrical impulse conduction along with an increase in ventricular action potential duration [83].

After the inoculation of ICs, we observed a decrease in the number of beats per minute at all tested time points, which could suggest an impairment of cardiac conduction, specifically in the sinoatrial node. The bradycardia observed may result from an alteration in the autonomic nervous system, with a parasympathetic predominance, either due to a direct effect on the neurons constituting this autonomic system, due to depolarization from the increase in cytosolic calcium, as we have seen occur *in vitro,* in cells with which the vesicles interact [70] or due to the permeabilization induced by EVs, allowing macromolecules to pass through the cytoplasmic membrane, as previously observed *in vitro.*

Finally, bradycardia may occur as a result of cell activation and consequent expression of chemokines involved in cardiac function due to the direct interaction between the immune response and the sympathetic nervous system (SNS) which is known to play an essential role in cardiac alterations [84–87]. It is known that an inhibition of cellular electrical activity occurs after treatment with TNFα, which is one of the cytokines predominantly expressed in myocardial tissue following the stimulation with EVs and ICs in our study [85]. This would confirm the role that certain inflammatory cytokines (IFNγ, TNFα, or CCL3) play in the induction of cardiac pathologies (cardiomyopathy and QTc prolongation) in the disease [88–90].

The bradycardia observed may predispose to heart failure due to reduced cardiac output and the dilation of the cardiac chambers. The differences found between the effects of EVs and ICs inoculation could be due to the ability of ICs to bind to cells, such as macrophages, that have specific receptors (FcγR) on their cell membrane for the Fc fraction of IgGs, thereby allowing ICs to bind to the cell membranes through these receptors and facilitating their uptake (opsonization). This binding may occur not only via FcγR receptors on macrophages but also through those present in other cells, including cardiomyocytes [91],

The population of resident cardiac macrophages represents approximately 6–8% of the non-cardiomyocyte population in the healthy adult mouse heart [92]. There are several populations of cardiac macrophages with distinct origins [93–95]. It has been described that most macrophages are located within the cardiac tissue as a constitutive component rather than as a result of an immune response induced by a pathogen. Cardiac macrophages participate in the heart reparation [96,97] and production of connexins, regulating contractile synchronization, but they are also involved in specific immune responses such as the phagocytosis of cellular debris, the recruitment of neutrophils, and the innate immune response against pathogens by participating in cytokine production [98].

Connexin 43 (Cx 43) is a protein located in the plasma membrane and the inner mitochondrial membrane. It oligomerizes into hexameric structures to form hemichannels, which pair with hemichannels on adjacent cells to create intercellular junctions called gap junctions. These structures facilitate electrical coupling and the direct exchange of signaling molecules—including second messengers, ions, ATP, NAD ⁺ , glucose, oxygen, and carbon dioxide—between cells. Under certain physiological and pathological conditions, such as inflammation, Cx43 hemichannels can also mediate the release of cyclic GMP-AMP (cGAMP), which activates the STING pathway, a key component of innate immunity responsible for detecting cytosolic DNA and triggering interferon production in response to pathogens [99,100]. Cx43 also participates in the regulation of cardiac fiber contraction, as the proper flow of ions and metabolites between cardiac myocytes contributes to synchronized contraction and signal exchange throughout cardiac tissue. Cx43 is expressed by both cardiomyocytes and intracardiac macrophages and, as noted, plays a role in regulating contractility between cardiac fibers. Suppression of Cx43 in macrophages delays atrioventricular conduction, and the ablation of Cd11bDTR macrophages in mice induces alters the cardiac rhythm due to atrioventricular block [101]. Our results show decreased expression of Cx43 (at both mRNA and protein levels) in mice inoculated with EVs and ICs compared to control mice. This could correlate with the cardiac rhythm alterations observed after ICs inoculation. A reduction in Cx43 synthesis was previously described by Adesse et al. in 2008 in cardiomyocytes infected *in vitro*, where cells during the intracellular development of *T. cruzi* reduced Cx43 expression compared to expression levels in control cells [102].

Barreto et al. (2024), recently showed that *T. cruzi*-infected mice developed impaired cardiac function consistent with chronic Chagasic cardiomyopathy (CCC) at 5 and 12 months post-infection, characterized by increased inflammation, fibrosis, and overexpression of IL-1β, TNF, and IFN-γ as well as an altered localization and phosphorylation of Cx43, with reduced colocalization compared to uninfected controls. Similar patterns were observed in human CCC heart samples. Stimulation of human cardiomyocytes or H9c2 cells with IL-1β, TNF, and IFN-γ reproduced these alterations in Cx43 distribution [103]. Other infections, such as HAdV-5, also disrupt Cx43 function via E4-mediated β-catenin activation, contributing to arrhythmias [104,105]. Likewise, SARS-CoV-2 S1 protein modulates Cx43 expression and enhances hemichannel activity in an ACE2-dependent manner, affecting ATP-mediated calcium signaling [106,107].

Cx43 also plays a key role in immune cells. Its deletion in astrocytes and alveolar macrophages increases proinflammatory cytokine secretion [108,109]. In contrast, anti-inflammatory cytokines like TGF-β1 and IL-10 upregulate Cx43 at macrophage–myocyte contact sites [110,111]. ATP released through Cx43 hemichannels activates the NOD-like receptor protein 3 (NLRP3) inflammasome and promotes IL-1β/IL-18 secretion, a process amplified by TLR2/4 stimulation. This ATP constitue an important DAMP (purinergic signaling molecule) that participates in the regulation of inflammation progression [112,113].

Studies on *T. cruzi* EVs reveal that only those from certain virulent strains, the most virulent ones, induced nitric oxide (NO) and TNF-a in a TLR2-dependent manner and that the strain’s DTU does not always correlate with this capacity [66]. Oliveira et al 2010 demonstrated that TLR-4 signaling is required in animals infected by *T. cruzi* for optimal production of IFN-ɣ, TNF-α and NO, interleukins that increase their expression together with NO in our results [114].

As mentioned above, according to our previous studies showing that EVs from trypomastigotes induce a rapid increase in cytosolic Ca^2^⁺ (as early as 5 min), accompanied by actin depolymerization and vimentin disorganization. Elevated Ca^2^ ⁺ levels, which inhibit Cx43 channel function [115]. may contribute to the observed effects. These changes likely involve modulation of Rho-GTPase pathways. Specifically, RhoA—known to inhibit Cx43 hemichannel activity —is downregulated within 30 min of EV interaction, more so than in direct parasite-cell interactions, highlighting the role of EVs in signaling modulation [116]. In this regard, we have reported that the expression of Rho-GTPases, particularly RhoA, decreases within 30 minutes of interaction in cells infected with *T. cruzi*, as well as in cells exposed to the parasite’s EVs. This reduction was sustained for up to 4 hours post-interaction. Notably, the decrease in RhoA expression was consistently more pronounced following interaction with EVs compared to direct infection with the parasite, suggesting a significant role for *T. cruzi*-derived EVs in modulating host cell signaling pathways [71].

The correlation between alterations in Cx43 expression and distribution in myocardial diseases, such as hypertrophic cardiomyopathy, heart failure, and ischemia, was described by Velia MP et al. in 2015 [117].

The ECG measurements results showed an increase in the PR interval which suggests an impairment in the conduction tissue, mainly in the atrioventricular node, which may indicate a first-degree atrioventricular block. This predisposes to asynchrony between atrial and ventricular contraction (electromechanical decoupling), resulting in reduced cardiac output and exacerbating heart failure [118,119]. The ECG alterations observed after the initial intravenous inoculations demonstrate the role of EVs as modulators of cardiac rhythm, suggesting an influence on the cytoskeleton of cardiac contractile cells, an alteration of ionic flows, or a modification of the functionality of nerve cells involved in cardiac contraction. The inoculation of ICs would simulate more advanced stages of the infection, either the late acute phase or the chronic phase of the disease, in which the presence of specific antibodies (mostly IgG2, in patients with CD) would naturally lead to the formation of ICs [58]. As observed in the results obtained, the intravenously inoculated EVs in animals are capable of inducing a response of specific antibodies that can recognize the antigens of these EVs in the bloodstream (Fig 2).

Vascular cell adhesion molecule-1 (VCAM-1) is a protein involved in the adhesion and migration of leukocytes during inflammation. It is expressed on the endothelial cells of blood vessels in response to inflammatory interleukins or TNFα, interacting with leukocyte integrins to facilitate their migration and adhesion to the endothelium, which enables the subsequent transmigration of these lymphoid cells [120]. The expression of VCAM-1 has been proposed as a biomarker in immunological diseases (experimental autoimmune myocarditis), as a predictor of mortality and morbidity in patients with chronic heart failure, and in those with endothelial injury in patients with coronary artery disease and arrhythmia. Similarly, an increase of VCAM-1 has been described in the cardiac stages of chronic Chagas Disease, leading to its proposal as a potential biomarker of disease progression [121–124]. Previously, an increase in the expression of VCAM-1 was described as a result of experimental infection in cell cultures by *T. cruzi* [125]. However, our results, obtained through immunochemical studies with confocal microscopy on heart sections, show a decrease in VCAM-1 expression levels in mice inoculated with both EVs and ICs compared to control mice. Vaitkevicius-Antão V et al. (2025) found a significant decrease in the levels of VCAM-1 in chronic patients who progressed from the indeterminate form to mild chronic Chagas cardiomyopathy. However, as the disease progressed to the symptomatic stages of cardiomyopathy, VCAM-1 levels approached the concentrations observed in the indeterminate group. These same authors suggest that the initial reduction in circulating VCAM-1 fragment levels, observed between the indeterminate and mild cardiac groups, may be related to the inflammatory response associated with the Th1 profile, particularly the production of NO, which suppresses the expression of adhesion molecules involved in cell migration and tissue infiltration [124,126]. An increase in the expression of interleukins corresponding to a Th1 inflammatory response, along with greater NO production, was observed in myocardial cells under our experimental conditions.

The expression of other proteins such as tubulin or dynein showed lower expression levels than the controls. The idea that cytoskeletal alterations may play a fundamental role in cardiac remodelling is increasingly becoming an important aspect of understanding cardiac function [127]. Cardiac myocytes are highly structured muscle cells that feature dense cytoskeletal networks organized into two groups: the contractile cytoskeleton formed by filamentous actin-myosin bundles making up the myofibrils, and the non-sarcomeric cytoskeleton, composed of β- and γ-actin, microtubules, and intermediate filaments. Microtubules and intermediate filaments form a reticulated scaffolding, being responsible for intracellular cargo transport, mechanical signal transmission, membrane system shaping, and the organization of myofibrils and organelles. In heart failure onset, the proliferation and post-translational modification of the microtubule network are linked to dysregulated processes, including the mechanical impairment of contraction and relaxation of cardiac myocytes [128].

It is well-known that alterations in the density of microtubules disrupt K^+^ ion flow in cardiac myocytes, which can lead to arrhythmias [129]. On the other hand, as previously mentioned, Cx43 is involved in the contractility of cardiac fibers and directly interacts in gap junctions with microtubules [130]. The expression levels of Cx43, as observed, decreased in treated mice, which, along with the reduced expression of tubulin, would contribute to relaxation of the heart and its consequent cavities increase and heart rate decrease. In this regard, as we have seen previously, the processes of actin depolymerization induced by *T. cruzi* EVs on the cells they interact with, together with the increase in Ca ^2+^ would promote the disorganisation of the cytoskeleton, would promote low levels of Cx43 expression in cardiomyocytes and thereby the lack of connectivity between muscle fibres and relaxation of the heart muscle [115,131].

Dynein is associated with tubulin microtubules, facilitating transport through the microtubules [128]. Furthermore, dynein is involved in the regulation of K^+^ channels [132]. K^+^ is necessary for cardiac functionality, and both the gain and loss of function of K^+^ channels in ventricular and atrial tissue can lead to arrhythmias or even cardiac arrest. Increased presence of potassium channels is associated with impaired heart rhythm (longer QT intervals), leading to the development of potentially fatal arrhythmias and ventricular fibrillation. Similarly, downregulation of K^+^ channels in heart failure also increases the risk of sudden cardiac death [133,134]. In some cases, alterations in heart rhythm, especially those referred to as inflammatory channelopathies, are determined by the elevation of an inflammatory response that directly modifies the function of ion channels present in cardiac cells [135,136], Where the activation of connexin 43 hemichannels allows the free exchange of potassium (K^+^) and calcium (Ca^2+^) ions between the cells, which plays a key role in modulating the inflammasome through NLRP3 and thus the inflammatory response [137].

In this way, TNFα, IL-1β, and IL-6 directly affect the function of cardiac ion channels [135,138,139]. TNFα induces dysfunction of gap junctions in atrial myocytes through alterations in the expression and/or distribution of Cx40 and Cx43, favoring slow and heterogeneous conduction in the atria [140]. Similarly, the increase of IL-1 affects K^+^ channels [138], and IL-6 increases the Ca^2+^ current and inhibits the rapid activating repolarizing K^+^ current through a pathway that involves the IL-6 receptor inducing heart rhythm disturbances [141].

The analysis of the longitudinal sections of the hearts of animals treated either with EVs or ICs showed an increase in the mean area of the right and left ventricles in animals following the inoculation of EVs and ICs, respectively, indicating an enlargement of the cardiac cavities, as well as a significant thinning of the wall in both ventricles and the intracardiac septum compared to the control. Other pathological manifestations such as myocarditis, vasculitis, immune cell infiltration, or fibrosis, evaluated by collagen production, were not observed in the microscopic examinations. All these cardiac alterations are described in CD-infected animals, similar to those observed in human pathology [5,142]. The absence of these pathological manifestations may be due to the shorter study period compared to those where such manifestations appear, which in all cases exceed 3 months post-infection, and where the contact time with the parasite or its derived products was much longer than in our experiments [18,103,143–145], which lasted 21 days with a total of 7 intravenous injections of EVs (equivalent to 2.8 x 10^7^ EVs per mouse/inoculation).

Despite not showing fibrosis and only a moderate dilation of the cavities, the presence of brain natriuretic peptide (BNP) in the mice serum is indicative of the damage induced by the EVs or ICs [146]. BNP plays a role as a reliable predictor of systolic and diastolic dysfunction of the left ventricle and is considered the most robust predictor in prospective studies of patients with Chagas Cardiomyopathy [22]. BNP is a peptide produced predominantly by the cardiac ventricles and to a lesser extent by the atria, increasing in response to the enlargement of the heart cavities. BNP levels rise in response to the dilation of ventricular myocytes or increased ventricular wall tension, as occurs in congestive heart failure (CHF) [122,147,148]. Similarly, it has been shown that BNP expression is induced in the presence of a Th1 immune response, specifically in the presence of IFNγ [149].

The EVs shed by *T. cruzi* contain components (GPI, DNA, and RNAs) that activate the different TLRs present in the various cells that make up cardiac tissue. The ability to activate cardiomyocytes during the innate response has been demonstrated both *in vitro* [150] and *in situ*, showing the production of interleukins by these cells [151]. The inflammatory cytokines produced following the stimulation of cardiomyocytes could be the cause of the cardiac damage that occurs in chronic Chagas cardiomyopathy (CCC) [152]. Among the major molecules present on the surface of the parasite’s EVs are transialidases, including proteins belonging to the seven families classified by Freitas [49,153]. These enzymes, capable of capturing sialic acid from the host’s glycoproteins and transferring it to the parasite’s mucins, may be one of the vesicular components responsible for triggering cytoskeletal modifications [154], increased intracellular calcium levels [155], and activation of NF-κB [156]. This leads to an inflammatory response through IL-6 release [157], activation of RORγt receptors and induction of a Th17 response [158] as well as the production of NO and ROS, all of which are associated with CD pathology [155].

Cardiomyocytes contain TLRs both on the membrane (TLR2 and TLR4) and inside (TLR3 and TLR9). These receptors stimulate the interleukin response and act through signaling mechanisms mediated by MyD88, among others [159–161]. Boyd et al. described how stimulation of TLRs by cardiomyocytes induces a decrease in their contractile capacity by activating an inflammatory response [162] also modulated through Cx43 hemichannels [163]. This, along with intracardiac macrophages that have TLRs capable of being activated by protein, lipid, and nucleic acid components present in EVs, modifies the cardiac cytokine profile [164]. Our results show that the interleukin profile constitutes an inflammatory profile, where *IL-1β*, *IFN-*γ, and *TNF-α* exhibit the highest expression levels, along with the enzymes responsible for inducing the production of nitric oxide (NO), leading to the subsequent generation of superoxide ions. The role of nitric oxide in CD is contradictory: on one hand, it has been correlated with a protective role against the parasite [165], while on the other hand, it has been associated with the contribution to cardiac damage [124,126,166]. The highest expression profiles of this inflammatory response are observed after the inoculation of the ICs, compared to the expression levels produced by the EVs, except in the case of IFNγ, in which treatment with EVs resulted in higher values than those induced by ICs. This profile is similar to that obtained in peritoneal macrophages after stimulation of mouse peritoneal macrophages [69]. The expression levels of *IL-12* after stimulation with EVs do not correspond to those shown by *IFN*γ, which could be explained by an independent expression pathway of IFNγ that is not typical of a Th1 response but rather depends on stimulation produced by IL-12. It is noteworthy that the low levels of *IL-6* expression induced by the ICs do not align with data that have been described experimentally *in vivo* in animals, similar to those observed in humans infected by the parasite [167–169]. IL-15 is a pleiotropic cytokine suggested to have a protective effect in myocarditis by reducing cardiomyocyte death and improving cardiac function [170]. In our study, *IL-15* shows an expression profile similar to that obtained in the peritoneal cavity, where it induced expression levels higher than those induced by the EVs or the control [69]. Th1 regulatory interleukins and markers of a Th2 response, such as *IL-4* and *IL-13*, showed lower expression levels compared to the control. However, *IL-10* and *TGFβ* exhibited higher expression levels than the control, with the ICs inducing the highest expression level in cardiac tissue.

Ba et al. (2010) studied the induction of cytokines and ROS in *T. cruzi*-infected cardiomyocytes, demonstrating that the elevated inflammatory response induced in the cells by parasitism (TNF-α and IL-1β) is mediated by the increase of superoxide radicals produced in the parasitized cells. The induction of a Th1 cytokine profile similar to that observed in CD cardiac patients, suggests that the interaction of EVs or ICs with the various cells that comprise the myocardial tissue can trigger both physiological and inflammatory cellular changes, leading to cardiac alterations in the different phases of Chagas Disease [67,150]. On the other hand, Smyth et al. found that oxidative stress induced by H₂O₂ alone was sufficient to alter the localization of Cx43 and significantly reduce its relative expression levels in cardiomyocytes, which explains the relationship between heart rhythm disturbances and inflammatory stress mediated by the immune response and inflammatory interleukins in particular [171].

The role of EVs of infectious forms of *T. cruzi* in cardiac pathology encompasses the three theories of cardiac damage proposed in the literature for CD: a) the persistence of parasitic material in the heart even in the absence of visible parasites, b) the secretion of products by the parasite that act on the heart’s contractile conduction system, and c) the inflammatory immune response that accentuates the development of cardiac pathologies without affecting the parasitic load.

## Conclusion

**Fig 8:**
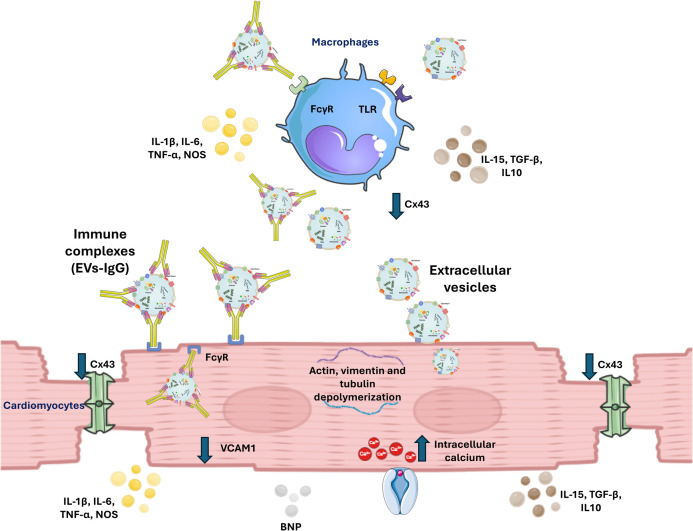
Summary figures show the cellular alterations induced in the mouse heart after 21 days of inoculation with EVs and ICs, which could be related to the pathological manifestations of Chagas disease cardiomyopathy. Figure created using Smart.Servier.com, which provides open-source images under the Creative Commons License 4.0.

In conclusion, the 21-day intravenous inoculation of *T. cruzi* EVs and their ICs was not capable of inducing the appearance of autoantibodies against host tissues in our experimental conditions, but it led to significant cardiac alterations in mice. Electrocardiographic changes, including modifications in heart rate and PR intervals, were observed, indicating disruptions in cardiac conduction. Histological analyses revealed alterations in the areas of heart cavities and thinning of the ventricular walls. There was a marked reduction in the expression of essential proteins for cardiac function, tubulin, and dynein, and in particular, connexin 43, which is necessary for the electrical connectivity between cardiac fibres. The decreased expression of VCAM-1 similar to that observed in patients in the transition from indeterminate form to mild chronic Chagas cardiomyopathy prevents the immune cell infiltration into the heart tissue and was associated with an altered cytokine expression profile. The elevated serum levels of BNP in EV- and IC-inoculated mice suggest the onset of cardiac damage (Fig 8). In this study, the changes observed after IC inoculation were more pronounced than those induced by EVs. Given that ICs are known to be present in the serum of chronic patients, this suggests that these alterations could accumulate over the years that the disease lasts, highlighting the potential role of *T. cruzi* EVs and their immune complexes in the pathogenesis of cardiac dysfunction in Chagas cardiomyopathy. Our study provides new insights into the mechanisms underlying the disease and may contribute to the development of innovative diagnostic and therapeutic strategies focused on EVs and ICs.

## Supporting information

S1 Fig Graphical abstract of the experimental design, indicating the moments of the interventions and experiments carried out. Figure created using Smart.Servier.com and OpenAI’s free ChatGPT platform, both of which provide open-source images under the Creative Commons Attribution 4.0 International License (CC BY 4.0).
(TIF)

S2 Fig Isolation of EVs shed by trypomastigotes of *T. cruzi* Pan4 strain. **(A)** transmission electron microscopy image of EVs (scale bar: 500 nm); **(B)** Measurements using ImageJ of the diameters of the EVs studied using electron microscopy. The graph shows the distribution of the different measurements in a scatter plot, indicating the mean diameter (85.7 + /− 9.2 nm) **(C)** Nanoparticle tracking analysis size distribution of EVs (mode size: 91.7 + /− 0.6 nm; mean size: 122.8 + /− 1.3 nm); **(D)** Western blot analysis for the confirmation of the presence of *T. cruzi* proteins in these EVs.
(TIF)

S3 Fig Antigenic recognition of a trypomastigote lysate by the sera of the different experimental mouse groups was assessed by indirect ELISA. The recognition of *T. cruzi* trypomastigote antigens correlates with the humoral response induced against the EVs shown in Fig 2. The values represent the mean ± SEM, with significance set at p < 0.05(*). Cut-off value = Mean of the PBS-injected mice absorbance + 3xSD.
(TIF)

S4 Fig (A) Analysis using anti-total mouse immunoglobulins as the secondary antibody showed that none of the mouse groups (EVs, ICs, and PBS-injected mice) developed autoantibodies against the mouse heart extract (blue) or the β1-adrenergic receptor (red), which is known to be present in cardiac tissue. Likewise, the anti-T. cruzi serum used to form the immune complexes did not show recognition of self-antigens. (B) To assess the avidity of antibody recognition, a 0.5 M KSCN incubation was performed after the primary antibody incubation; however, no statistically significant differences were observed.
(TIF)

S5 Fig Picrosirius Red-stained slides of heart samples from mice injected with EVs (green) or ICs (blue) showed no difference in the presence of collagen fibers when compared to PBS-injected mice (red). (**A**) Images acquired by dipolarized light microscopy did not suggest variations in the collagen content or collagen deposits. (**B**) Signal intensity was quantified using ImageJ, and the values were referred to as the intensity present in the controls (PBS). The values represent the mean ± SD.
(TIF)

S1 Table Sequences of the forward and reverse primers employed for gene amplification and expression analyses. (DOCX)

S2 Table QTc data (ms) obtained from the ECG analysis. The sample size for each group was 5 mice.
(DOCX)
