# Supplementary figures and images for: Cardiac alterations induced by Trypanosoma cruzi extracellular vesicles and immune complexes
Source: PLoS Negl Trop Dis. 2025 Jul 7;19(7):e0013273. doi: 10.1371/journal.pntd.0013273 (PMC12251207; doi:10.1371/journal.pntd.0013273)

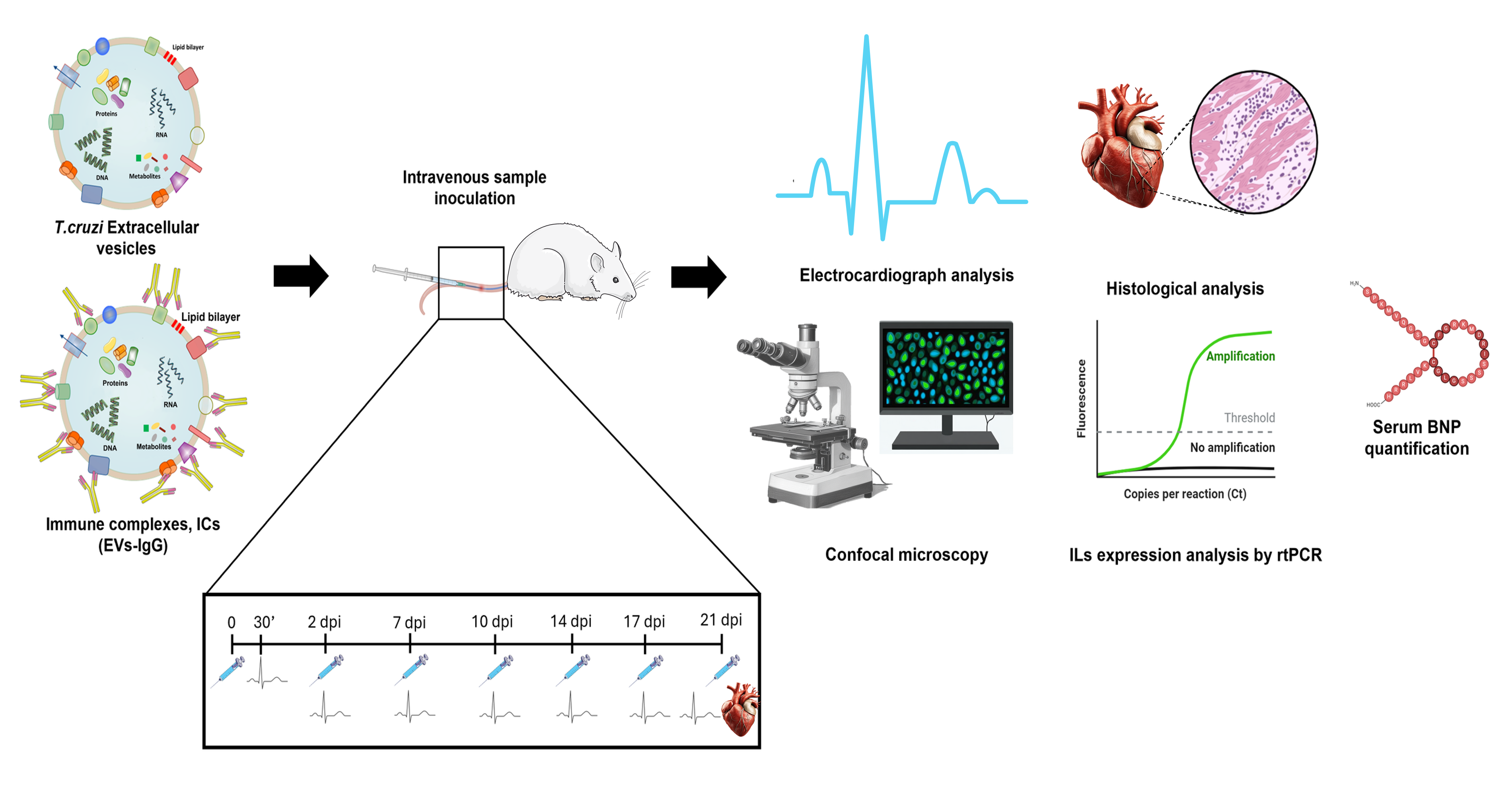

Supplement: S1 Fig — Figure created using Smart.Servier.com and OpenAI’s free ChatGPT platform, both of which provide open-source images under the Creative Commons Attribution 4.0 International License (CC BY 4.0). (TIF) [file pntd.0013273.s001.tif]

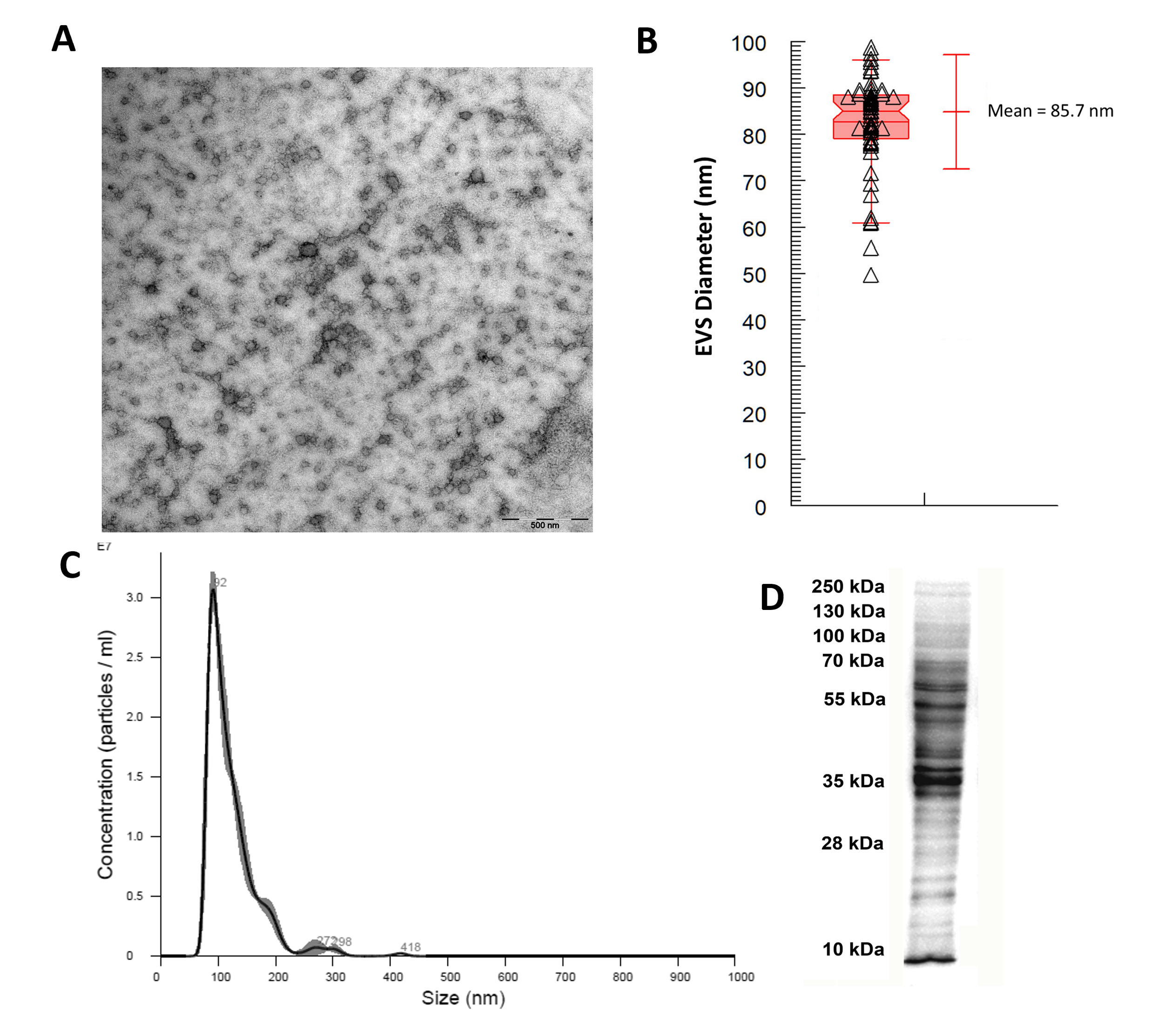

Supplement: S2 Fig — (A) transmission electron microscopy image of EVs (scale bar: 500 nm); (B) Measurements using ImageJ of the diameters of the EVs studied using electron microscopy. The graph shows the distribution of the different measurements in a scatter plot, indicating the mean diameter (85.7 + /− 9.2 nm) (C) Nanoparticle tracking analysis size distribution of EVs (mode size: 91.7 + /− 0.6 nm; mean size: 122.8 + /− 1.3 nm); (D) Western blot analysis for the confirmation of the presence of T. cruzi proteins in these EVs. (TIF) [file pntd.0013273.s002.tif]

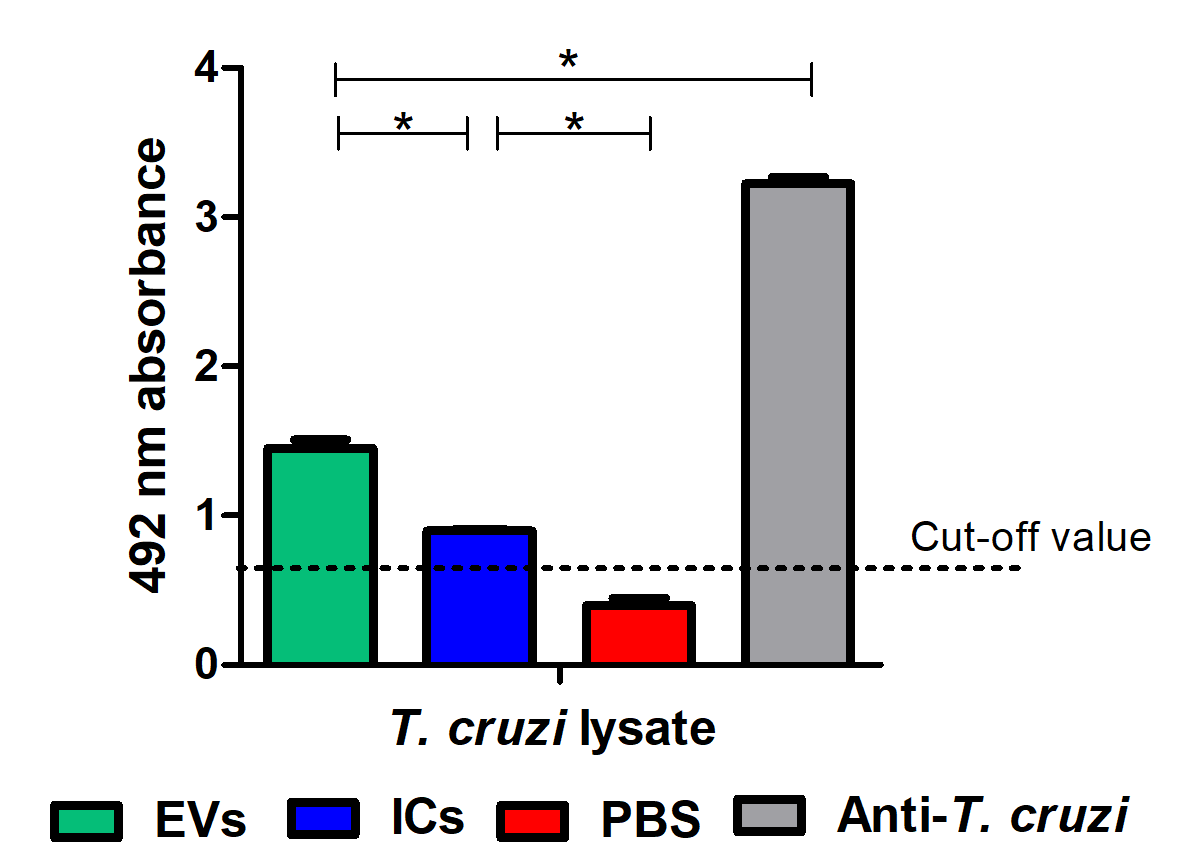

Supplement: S3 Fig — The recognition of T. cruzi trypomastigote antigens correlates with the humoral response induced against the EVs shown in Fig 2. The values represent the mean ± SEM, with significance set at p < 0.05(*). Cut-off value = Mean of the PBS-injected mice absorbance + 3xSD. (TIF) [file pntd.0013273.s003.tif]

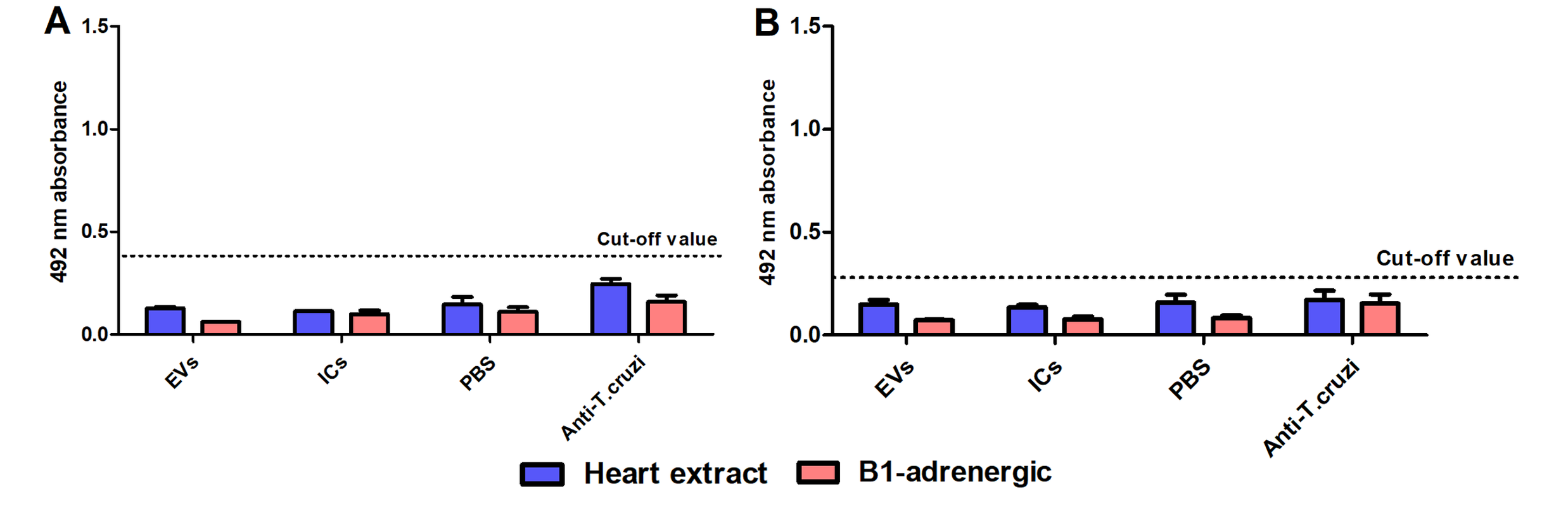

Supplement: S4 Fig — Likewise, the anti-T. cruzi serum used to form the immune complexes did not show recognition of self-antigens. (B) To assess the avidity of antibody recognition, a 0.5 M KSCN incubation was performed after the primary antibody incubation; however, no statistically significant differences were observed. (TIF) [file pntd.0013273.s004.tif]

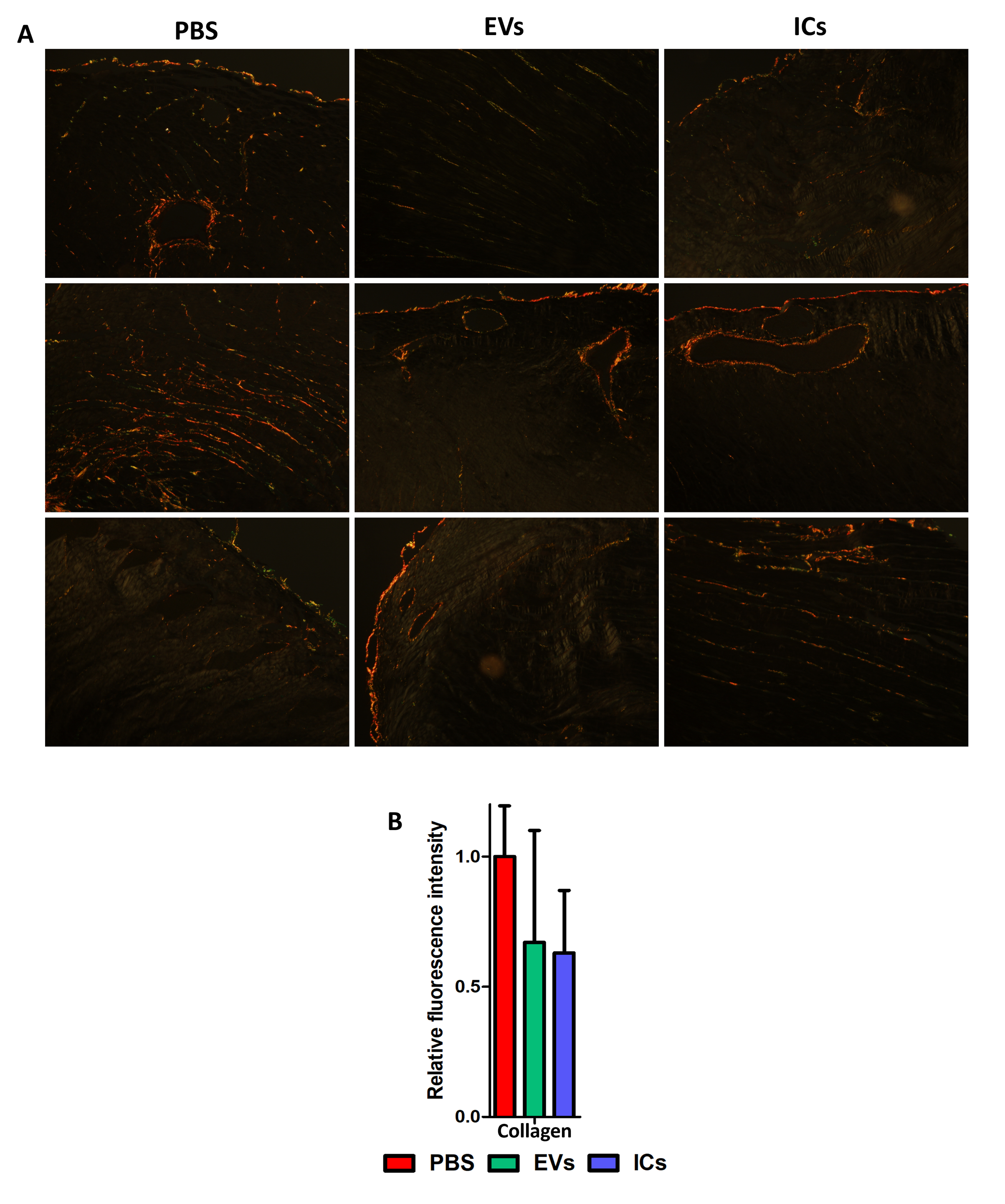

Supplement: S5 Fig — (A) Images acquired by dipolarized light microscopy did not suggest variations in the collagen content or collagen deposits. (B) Signal intensity was quantified using ImageJ, and the values were referred to as the intensity present in the controls (PBS). The values represent the mean ± SD. (TIF) [file pntd.0013273.s005.tif]
