# Supplementary material for: Cardiac alterations induced by Trypanosoma cruzi extracellular vesicles and immune complexes
Source: PLoS Negl Trop Dis. 2025 Jul 7;19(7):e0013273. doi: 10.1371/journal.pntd.0013273 (PMC12251207; doi:10.1371/journal.pntd.0013273)
Supplement: S1 Table — (DOCX) [file pntd.0013273.s006.docx]

**Table S1**. Sequences of the forward and reverse primers employed for gene amplification and expression analyses.

| **Gene** | **Forward sequence (5'-3')** | **Reverse sequence (5'-3')** |
| --- | --- | --- |
| **IL-1β** | TGCCACCTTTTGACAGTGATG | CTCTTGTTGATGTGCTGCTG |
| **IL-12** | GACCAAACCAGCACATTGAA | CTACCAAGGCACAGGGTCAT |
| **IFNγ** | CACCCTGAAGTCGTTGTGAA | GATCTCCCCACTCCGGTTAT |
| **IL-6** | AGTTGCCTTCTTGGGACTGA | TCCACGATTTCCCAGAGAAC |
| **TNFα** | CCCCAAAGGGATGAGAAGTT | CACTTGGTGGTTTGCTACGA |
| **IL-15** | CATTTTGGGCTGTGTCAGTG | TGCAACTGGGATGAAAGTCA |
| **NOS** | TGACACACAGCGCTACAACA | CCATGATGGTCACATTCTGC |
| **IL-4** | CCTCACAGCAACGAAGAACA | ATCGAAAAGCCCGAAAGAGT |
| **IL-13** | AGCATGGTATGGAGTGTGGA | TTGCAATTGGAGATGTTGGT |
| **TGFβ** | TGGAGCAACATGTGGAACTC | AGCCTTGTATCCCGTCTCTT |
| **IL-10** | CAGAGCCACATGCTCCTAGA | TCATTTCCGATAAGGCTTGG |
| **Cx43** | AAAGTGGTGGAACTCCTTGG | GAGCGAGAGACACCAAGGAC |
| **GAPDH** | ATGTGTCCGTCGTGGATC | ACCTGGTCCTCAGTGTAGC |
