# Supplementary material for: Cardiac alterations induced by Trypanosoma cruzi extracellular vesicles and immune complexes
Source: PLoS Negl Trop Dis. 2025 Jul 7;19(7):e0013273. doi: 10.1371/journal.pntd.0013273 (PMC12251207; doi:10.1371/journal.pntd.0013273)
Supplement: S2 Table — The sample size for each group was 5 mice. (DOCX) [file pntd.0013273.s007.docx]

**Table S2**. QTc data (ms) obtained from the ECG analysis. The sample size for each group was 5 mice.

| **Time** | **30 min** | | | |
| --- | --- | --- | --- | --- |
| **Sample** | **Preinoculum** | **PBS** | **EVs** | **ICs** |
| **Mean** | 0,99 | 1,05 | 1,10 | 0,97 |
| **SD** | 0,10 | 0,29 | 0,17 | 0,12 |
| **Time** | **2 days** | | | |
| **Sample** | **Preinoculum** | **PBS** | **EVs** | **ICs** |
| **Mean** | 0,99 | 0,89 | 1,07 | 1,07 |
| **SD** | 0,10 | 0,16 | 0,26 | 0,23 |
| **Time** | **7 days** | | | |
| **Sample** | **Preinoculum** | **PBS** | **EVs** | **ICs** |
| **Mean** | 0,99 | 0,89 | 1,08 | 1,09 |
| **SD** | 0,10 | 0,27 | 0,18 | 0,25 |
| **Time** | **10 days** | | | |
| **Sample** | **Preinoculum** | **PBS** | **EVs** | **ICs** |
| **Mean** | 0,99 | 1,11 | 0,96 | 1,09 |
| **SD** | 0,10 | 0,32 | 0,26 | 0,21 |
| **Time** | **14 days** | | | |
| **Sample** | **Preinoculum** | **PBS** | **EVs** | **ICs** |
| **Mean** | 0,99 | 1,10 | 1,15 | 1,07 |
| **SD** | 0,10 | 0,33 | 0,28 | 0,13 |
| **Time** | **17 days** | | | |
| **Sample** | **Preinoculum** | **PBS** | **EVs** | **ICs** |
| **Mean** | 0,99 | 1,10 | 1,08 | 1,19 |
| **SD** | 0,10 | 0,35 | 0,21 | 0,26 |
| **Time** | **21 days** | | | |
| **Sample** | **Preinoculum** | **PBS** | **EVs** | **ICs** |
| **Mean** | 0,99 | 1,06 | 0,97 | 1,04 |
| **SD** | 0,10 | 0,37 | 0,28 | 0,26 |
